# Efficient joint resource allocation using self organized map based Deep Reinforcement Learning for cybertwin enabled 6G networks

**DOI:** 10.1038/s41598-025-02274-7

**Published:** 2025-06-05

**Authors:** Nivetha A, Preetha KS

**Affiliations:** School of Electronics Engineering, Vellore Institute of Technology, Vellore, Tamilnadu 632014 India

**Keywords:** Self-organized map, Cybertwin, Deep Reinforcement Learning, Wired + wireless (hybrid) networks, 6G, Electrical and electronic engineering, Computer science

## Abstract

Sixth-generation wireless communication has emerged, stimulating the rapid growth of numerous types of real-time applications that are characterized by their high data computing demands and formation of massive data traffic. Cybertwin-enabled edge computing has become a logical way to satisfy the enormous user demands. However, there are drawbacks to this advancement as well. The effective distribution of resources while balancing the demands for computing, communication, and caching is a major problem in edge networks. The resource allocation problem in dynamic edge computing systems is too complex to address with traditional statistical optimization techniques. Therefore, a Joint Resource allocation method using Self-Organized Map (SOM)-based Deep Reinforcement Learning (DRL) is proposed for cybertwin-enabled 6G wired + wireless (hybrid) networks. This approach controls the clustering capabilities of SOM to organize the state space, followed by the decision-making strength of RL to select optimal actions for resource allocation in dynamic and real-time environments. The objective is to minimize overall latency and energy consumption. From the results analysis, using SOM-DRL, the hybrid network model outperforms the wireless-only model in terms of latency and energy consumption than the existing MATD3 method by achieves 3.34% of energy consumption, 3.17% of latency, and 7.30% of completion time.

## Introduction

Recently, the Internet of Everything (IoE) has become crucial in supporting data-intensive applications such as smart healthcare, virtual reality (VR) gaming, and smart industries. These applications demand technological advancements and the evolution of communication systems beyond fifth-generation (5G) networks^1^. The advancements in the Internet of Things (IoT) and the proliferation of sensing devices have significantly contributed to the rapid expansion of data-intensive applications. Support the growing IoT ecosystem, connecting billions of devices seamlessly, from smart homes and cities to industrial IoT and connected healthcare. Real-time data processing and transmission are crucial for performance and user experience^2^. This connectivity facilitates the effortless collection, analysis, and sharing of big data across diverse platforms, creating a more integrated and responsive digital ecosystem^3^. The scale of this integration is substantial, with the International Telecommunication Union (ITU) projecting a massive increase in the number of IoT devices. By 2030, it is estimated that our digital ecosystem will encompass approximately 125 billion IoT devices, and these devices are expected to generate an astonishing 4395 Exa bytes of data annually. Hence the large volume of data must be managed, analyzed, and used^4^.

Users request latency is decreased by edge computing, which moves computation, communication, and caching tasks to the edge of the network. Moreover, these features are used by the edge-cloud collaboration architecture to improve quality of service (QoS) and user experience for applications that are delay-sensitive^5^. It is predicted that the 6G network topology based on cybertwin will shift from end-to-end to cloud-to-end connections. The Cyber Twin, functioning as a digital equivalent, improves the network’s scalability, flexibility, dependability, and security. In order to share 3Cs such as Computing, Caching, and Communication resources through a real-time multi-agent trading platform, a distributed cloud network operating system is described^6,7^. To enhance network performance by reducing delays and bandwidth consumption, edge computing and caching technologies are introduced in 6G-based cybertwin networks. However, limited edge resources and increasing task complexity pose challenges. Efficient resource utilization in communication, computing, and caching is crucial, but traditional methods still face issues. Artificial intelligence algorithms present effective ways to tackle these complicated joint resource allocation issues^8^.

### Motivation and objectives

The motivation of the work is described as follows:

(i) Traditional statistical optimization techniques struggle to handle the complexity and dynamic nature of edge computing environments, where the need for real-time, adaptive resource allocation is critical.
(ii) Cybertwin-driven network is an innovative way to manage resource allocation, but optimizing their performance in a hybrid wired + wireless network remains a challenge.
(iii) Reducing latency and energy consumption is crucial for improving the Quality of Experience (QoE) in real-time applications.
(iv) The integration of deep learning techniques, such as SOM and Deep RL provides a potential solution to deal with the resource allocation difficulties and improve overall network expertise, especially in the case of hybrid (wired + wireless) networks.

*Objectives* Resource allocation is a critical aspect of ensuring that wireless networks operate efficiently and reliably. It plays a key role in maintaining network stability by effectively distributing resources among competing demands. In the context of 5G and the emerging 6G networks, the complexity of resource allocation intensifies as diverse service requirements arise from various industries, each with its own unique needs. Meeting these demands will require advanced resource management scheme that can revise to the dynamic and heterogeneous nature of 6G networks.

### Contributions

The major contributions of the proposed hybrid SOM-DRL model for cybertwin-enabled 6G networks are:

- *Cybertwin-driven network design* Novel end-edge cloud architecture is developed, enabling user devices to offload compute-intensive tasks to the edge cloud or core cloud. The scheme efficiently manages 3C’s in order to minimize the latency and energy consumption while implementing a secure authentication mechanism for cybertwins to ensure only authorized digital representations operate in remote environments.
- *Joint resource allocation algorithm* The resource allocation complication is standardized as a Markov Decision Process (MDP) to address the complex demands of dynamic edge computing environments. A hybrid algorithm combining a Self-Organizing Map for state space clustering and a Deep RL Actor-critic model is proposed to optimize resource allocation, reducing both latency and energy consumption.
- *Performance evaluation* The effectiveness of the proposed hybrid SOM-DRL approach is validated through extensive simulations. Results demonstrate superior performance compared to traditional methods, like Random Resource Allocation (RRA), Greedy Resource Allocation (GRA), Multi-Agent Deep Deterministic Policy Gradient (MADDPG), and Multi-Agent Twin Delayed Deep Deterministic (MATD3) algorithm, achieving improved latency and energy efficiency in real-time applications for 6G networks.

### Outline of the work

The structure of the paper is organized as Section “Introduction” gives the introduction of 6G-based cybertwin; Section “Related works” reviews the related works on resource allocation; Section “System model” discusses the system model; Section “Hybrid SOM and deep RL-based cybertwin driven joint resource allocation” describes the proposed model which combines SOM for state space clustering and Deep RL Actor-critic model for optimization of resource allocation; Section “Results and discussion” discusses the experimental analysis for wireless and hybrid (wired + wireless) communication and Section “Conclusion” concludes the work and discusses the future work.

## Related works

Many computationally demanding applications have been developed as a result of the smart IoT device’s explosive proliferation. Yet, resource allocation at the network edge has become a typical difficulty due to constrained resources. Industry and academia are giving close attention to resource allocation at the edge, including caching, processing, and communication, in order to enhance user experience while minimizing latency. Numerous studies have been conducted, and resource allocation remains a critical issue in cybertwin-based 6G.

Mobile Edge Computing (MEC) has emerged to enhance cloud infrastructure survivability. Though many efforts have focused on efficient resource allocation for application offloading, most address either computational or communication protocols separately, without considering cooperative solutions^9^. Edge Computing (EC) was introduced to reduce latency by moving task processing closer to users. The edge is characterized by heterogeneous Edge Devices (EDs), making it essential to develop solutions that account for their varying physical resources. A resource representation scheme was proposed where each ED shares its resource information edge cloud. The resource data is provided whenever resource allocation is needed^10^.

The Cyber Twin technology is set to transform 6G networks by enhancing communication, resource allocation, and digital asset management with Artificial Intelligence (AI). It adjusts computing resource requests based on user demands and resource availability. Current architectures apply uniform AI settings, complicating dynamic resource management. An adaptive AI framework was introduced with efficient feature selection, optimizing AI model complexity and reducing overhead while maintaining accuracy for Cyber Twin-driven connected vehicles in 6G^11^. To optimize the handling of computation-intensive and time-sensitive tasks by User Terminal Devices (UTDs), MEC is utilized. Given varying UTD performance and MEC server resource constraints, this paper addresses the joint optimization of computation offloading and resource allocation in multi-user and multi-server scenarios. The Mixed Integer Nonlinear Programming (MINP) problem was modeled to minimize energy consumption and propose a two-stage genetic algorithm-based heuristic to iteratively update solutions, achieving stable convergence^12^.

Fog Radio Access Networks (F-RANs) support IoT services through edge caching and computing, but existing methods for computation offloading and resource allocation are inefficient and static. This can be addressed by formulating a joint problem of mode selection, resource, and power allocation to minimize latency. DRL-based scheme optimizes whether tasks are processed locally or offloaded, and allocates resources effectively based on the serving tier^13^. A Cyber Twin-driven edge framework utilizing 6G technology has been proposed with an intelligent service provisioning strategy to support large-scale IoE applications. The framework uses DRL to distribute tasks based on dynamic service requirements. Additionally, a classifier called Support Vector Machine (SVM) was employed at the edge network to interpret data and guarantee immense accuracy^14^.

MEC reduces mobile devices’ computational burden by offloading tasks to neighboring users. This work addresses the challenge of resource and channel allocation when numerous mobiles upload tasks to an MEC server in a single cell. In order to decrease the consumption of energy, an optimization problem was created using Greedy resource allocation and the Selection of Maximum Saved Energy First (SMSEF) technique^15^.

Multi-agent Deep Deterministic Policy Gradient is a multilevel task offloading approach that was developed for both delay-sensitive and delay-tolerant tasks. In order to execute this scheme, edge computing and AI were combined with a cybertwin-based network. This technique allows for decreased overhead, dynamic real-time resource allocation, and faster task processing. Furthermore, federated learning was used to effectively train the MADDPG model^16^. A multi-Unmanned Aerial Vehicle (UAV) cluster system was demonstrated, enabling the functions of resource distribution and offloading computational tasks. It is suggested that a multi-agent deep reinforcement learning^17^ technique be used to reduce total network computing costs while maintaining the QoS needs of IoT devices. The problem is formulated as an MDP-based stochastic game to optimize long-term computation cost in terms of energy and delay, considering UAVs’ time-varying channel strength and dynamic resource requests in the aerial-to-ground network.

A flexible AI scheme based on active feature selection was proposed to coordinate with cybertwin’s resource allocation, enabling the AI model to adaptively adjust its complexity based on available computing resources. This framework characterizes the collected impacts of feature combinations on AI modeling sequel and quantifies feature interactions using non-additive measures^11^. The combination of the wired and wireless links (hybrid communication) is discussed in^18^ to analyze the secrecy performance of hybrid communication in a cybertwin-based 6G network, which results in higher secrecy performance when compared with the wireless link.

An efficient task offloading in resource constrained Industrial IoT heterogeneous networks was addressed in^19^ for minimizing energy consumption and network delay. Here Hall’s marriage Theorem and a server selection algorithm were applied for matching feasibility to achieve good performance. Deep Q-Network (DQN) and on-policy (Asynchronous Advantage Actor-Critic (A3C)) Deep Reinforcement Learning (DRL) techniques were proposed for optimal resource utilization for maximizing the reward^20^. Efficient task offloading is crucial to balance demand and QoS needs. The role of edge computing in Industry 5.0 was surveyed, highlighting its importance and potential technologies. It covers architecture, key objectives, and research challenges like privacy, human–robot collaboration, sustainability, and network robustness^21^. The various design principles that support Industry 5.0's integration of AI and IoT have been explained. The need of employing Deep Reinforcement Learning (DRL) techniques for task offloading is emphasized^22^.

Even though resource allocation for edge computing has advanced significantly, there are still a number of issues with present techniques. Because edge environments are dynamic and complicated, real-time adaptive resource allocation is challenging using traditional statistical optimization techniques. Although cybertwin-driven networks present an appropriate resource management strategy, optimizing their performance in hybrid wired + wireless networks are still difficult. Additionally, current research frequently focuses on communication or computational optimization independently, but a comprehensive cooperative solution is missing. For real-time applications, lowering latency and energy usage is essential, but existing methods fall short of striking the ideal balance. Especially in heterogeneous 6G networks, the combination of Deep RL and self-organizing models offers a scalable, innovative way to improve network efficiency and adaptability.

## System model

**Fig. 1:**
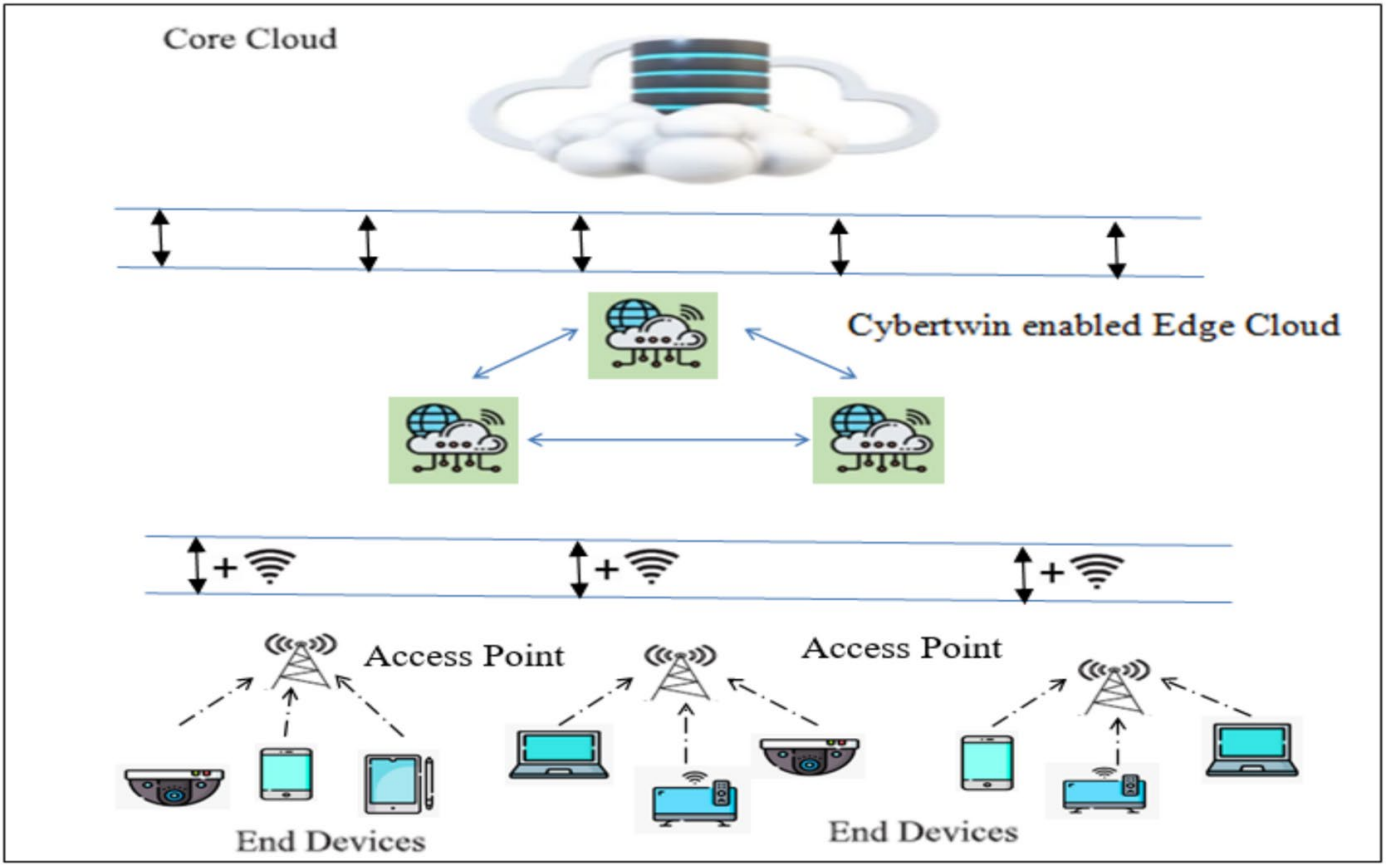
Hybrid cyber-twin-enabled 6G cooperative network architecture.

A cybertwin-enabled edge-end cloud network is analyzed, as depicted in Fig. 1, structured into three level hierarchies^23^. The lower layer has various end devices like smartphones, smart sensors, and smart vehicles, all supporting diverse applications such as video streaming, online gaming, augmented reality/virtual reality (AR/VR), and finding routes, each with distinct computational demands. While some applications are time-sensitive and demand real-time responses, others may be delay-tolerant with high processing requirements.

In this proposed model, the wired (Core cloud and cybertwin-enabled edge clouds) and wired + wireless communication between end devices and cybertwin-enabled edge cloud components were considered. Edge clouds are connected to the core cloud via wired links, while end devices connect to edge clouds by both wired and wireless links. All components, including edge clouds and end devices, communicate wired and wirelessly.

Let us assume a network that comprises of ‘N’ End devices (*E*_*D*_), and it is denoted by the set of *E*_*D*_ = (*E*_*D1*_*,E*_*D2*_*,…E*_*Di*_*,…E*_*DN*_). Assume that these devices can able to produce a maximum of one computation task at each time slot ‘t’ according to the definition *E*_*Di*_*(t)* = {*C*_*i*_*(t), I*_*i*_*(t), D*_*i*_*(t)*} where *C*_*i*_*(t)* represents computation cycles that is required to complete the task, *I*_*i*_*(t)* denotes the data size which is given as input for the task, and *D*_*i*_*(t)* represents the maximum allowable time limit for the task’s execution. These devices have the capacity to transfer computational tasks to nearby devices due to their restricted resources, ensuring that the user’s QoS requirements are met efficiently.

A network of edge clouds, each with several distributed edge servers, comprises the middle layer of the structure. While their resource capabilities are not as extensive as core clouds, these edge clouds are equipped with sufficient capacity to enhance the response time for end devices. Their close proximity to the *EDs* ensures that the applications that are delay-sensitive can meet their deadlines efficiently. Here, the network is considered with M number of edge clouds, and it is represented by the set *E*_*C*_ = (*E*_*C1*_*, E*_*C2*_*,…E*_*Cj*_*,…E*_*CM*_). Computing-intensive tasks can be assigned to the edge cloud by end nodes. The edge cloud may assign the terminal device responsibilities to a neighboring edge cloud for further processing if it becomes overloaded.

The higher layer consists of core clouds linked by high-speed optical lines (wired communication), offering 3C’s services. These clouds are perfect for processing computationally demanding and delay-tolerant tasks from end devices via the edge cloud because of their abundance of resources. By doing this, the load on edge servers decreases, consequently enhancing network performance overall. In comparison to typical cloud systems, application devices are not established close to core clouds.

In an *E*_*C*_, the cyber-twin serves as the end device’s digital twin, using its knowledge of device features to optimize task scheduling and resource allocation. It assists with communication, logs network data, and manages digital assets. Tasks from end devices are transmitted to cyber-twins, which analyze the requests and offload them to the appropriate edge or core cloud for execution. Once processed, the results are transmitted back to the terminal devices via the cyber-twin.

Cyber-twin collects the information status such as computational needs, task priority, task queue, rate of data transmission from the end devices and the available computing resources of the edge cloud. The cybertwin functions like a scheduler which helps in managing the tasks and resources more efficiently. End device execution can occur locally, be offloaded to a neighboring edge cloud, be transferred between edge clouds, or be offloaded to a core cloud for processing (categories of end device accomplishments).

### User mobility

It’s important to offer mobile users a long-lasting service solution, particularly in cases where an ED’s changing position might disrupt its link to its matching cyber twin. The mapping between the end device and its cybertwin needs to adjust for user mobility in order to preserve QoS. In order to access services, end devices use mobility management with a cyber-twin link to a cyber-twin that is located on an edge cloud. For maintaining effective services, the cyber-twin migrates to an alternate edge cloud when a user moves from the original cyber-twin. In this proposed work, the location of the user is determined using a mobility model similar to^24^, designed to track a pedestrian’s trajectory. The system operates in discrete time slots, *t* = {*t1, t2, t3,…*}, with each slot having an equal interval length ‘τ’. The position of a mobile device ‘E_D_’ at the time ‘*t*’ is expressed as *loci(t)* = *(x(E*_*Di*_*, t), y(E*_*Di*_*, t))*. If the device is stationary, the position remains constant, but for mobile devices, the location updates in each time slot. The trajectory only covers a modest distance in each time period since the humans are walking at a relatively slow speed. As a result, it is assumed that the user stays within a single edge cloud’s coverage region for a single time period. The distance to the hosted edge cloud can be calculated based on the location of the end device.

1 $$Dist_{a,b} \, = \,\sqrt {\left( {x_{a} \, - x_{b} } \right)^{2} \, + \,\left( {y_{a} \, - \,y_{b} } \right)^{2} }$$

where *x*_*a*_ and *y*_*a*_ are the coordinates of entity ‘a’ (e.g., an end device) and *x*_*b*_ and *y*_*b*_ are the coordinates of entity ‘b’ (e.g., an edge cloud). The end devices move within the defined area using a Random Waypoint mobility model, where each device travels toward a randomly selected waypoint at a constant speed.

### Computation and communication model

The cyber-twin will use the end device’s resources for computing when it decides to execute a task locally. If *f*_*i*_ is the CPU cycle frequency of the local end device *E*_*Di*_, then Eq. (2) can be used to express the total task execution latency,

2 $$T_{exec} = \frac{{C_{i} \left( t \right)}}{{f_{i} }}$$

The energy consumption is calculated by considering the distance parameter (if the tasks require real time updates and if the data is to be transferred to the nearby nodes or cybertwin servers) and CPU cycle frequency, and it is expressed in Eq. (3),

3 $$E_{Con} = Dist_{a,b} \cdot C_{i} \left( t \right) \cdot f_{i}$$

#### Task offloading from E_D_ to E_C_

With a device-to-device wireless link, the work to be performed on the edge cloud will be transferred to its target. The amount of the input task and the wireless channel conditions affect the communication’s transmission delay. The Shannon-Hartley theorem, which is provided in Eq. (4), can be used to calculate the maximum achievable data transmission rate utilizing a Time division multiple access (TDMA) communication paradigm between the E_D_ and the E_C_ with a total available bandwidth “B.”,

4 $$D_{{TR\left( {i,j} \right)}} \left( t \right) = B_{ij} \cdot log_{2} \left( {\frac{{P_{ij} \cdot g_{ij} \left( t \right)}}{{\sigma^{2} + {\upeta }_{ij} \left( t \right)}}} \right)$$

where *D*_*TR(i,j)*_*(t)* represents the maximum rate of data transmission between E_Di_ and E_Cj_ at time slot ‘t’. *P*_*ij*_ indicates the transmission power of *E*_*Di*_ and *E*_*Cj*_, and *σ*^*2*^ denotes noise power. Also, *g*_*ij*_*(t)* and *η*_*ij*_*(t)* indicate the interference and channel gain at the time ‘t’. A multipath fading and distance-dependent path loss model is used to assess the channel gain, which can be expressed using Eq. (5).

5 $$g_{i,j} \left( t \right) = \lambda \cdot \frac{1}{{Dist_{i,j} \left( t \right)}}\left| {\left| {h^{m} } \right|} \right|^{2} \zeta$$

Here *λ* denotes the coefficient of path loss, the distance between *E*_*Di*_ and *E*_*Cj*_ at time instance ‘*t*’ is given as *Dist*_*i,j*_*(t)*; Rayleigh fading coefficient is denoted as *h*^*m*^, and log-normal distributive shadowing is represented as ‘*ζ*’.

The transmission time from *E*_*Di*_ to *E*_*Cj*_, the task execution time at *E*_*Cj*_, and the time required for transmitting the result back to *E*_*Di*_ are all included in the overall end-to-end latency when a computation task is offloaded to the edge cloud for execution. The computed result’s minimal size, however, allows the result transmission time to be ignored. Equation (6) can be used to express the total delay for the offloaded process at *E*_*Cj*_ if *f*_*j*_ is the edge cloud’s CPU cycle frequency.

6 $$T_{i,j}^{edge} \left( t \right) = \frac{{I_{i} \left( t \right)}}{{D_{{TR\left( {i,j} \right)}} \left( t \right)}} + \frac{{C_{i} \left( t \right)}}{{f_{j} }}$$

The calculation of total energy consumption consists of the energy needed for both transmission and used during task execution. Therefore, Eq. (7) gives the total energy consumption.

7 $$E_{i,j}^{edge} \left( t \right) = \frac{{P_{i,j } \cdot I_{i} \left( t \right)}}{{D_{{TR\left( {i,j} \right)}} \left( t \right)}} + C_{i} \left( t \right) \cdot f_{j}^{2} \cdot {\upeta}_{j}$$

The data transmission rate for wired communication is calculated using Eq. (8), where *B*_*wired*_*(t)* indicates the available bandwidth of the wired link at time *t*; *P*_*wired*_*(t)* represents transmission power, $\sigma_{wired }^{2}$ indicates noise power.

8 $${D_{TR} }_{wired}^{i,j} \left( t \right) = B_{wired} \left( t \right) \cdot log_{2} \left( {1 + \frac{{P_{wired} \left( t \right)}}{{\sigma_{wired}^{2} }}} \right)$$

Therefore, the total data transmission rate for hybrid communication, which includes both wired and wireless links, is calculated by using Eq. (9),

9 $${D_{TR} }_{Effective}^{i,j} \left( t \right) = {D_{TR} }_{wired}^{i,j} \left( t \right) +{ D_{TR} }_{wireless}^{i,j} \left( t \right)$$

The total energy consumption, which includes energy required in hybrid (wired + wireless) transmission, as well as execution, can be represented using Eq. (10). Here, *P*_*i,j*_ denotes transmission power for the combined (wired + wireless) link, and *η*_*j*_ represents the constant computing coefficient of edge cloud *E*_*Cj*._

10 $$E_{i,j}^{edge} \left( t \right) = P_{i,j} \cdot \frac{{l_{i} \left( t \right)}}{{{D_{TR} }_{Effective}^{i,j} \left( t \right)}} + C_{i} \left( t \right) \cdot f_{j}^{2} \cdot {\upeta}_{j}$$

The latency for wired communication between end devices and the cyber twin-enabled edge cloud is calculated using Eq. (11). Here, R_wired_ represents the fixed data rate for the wired link and $l_{i} \left( t \right)$ denotes the size of the task.

11 $$T_{wired}^{{\left( {i,j} \right)}} \left( t \right) = \frac{{l_{i} \left( t \right)}}{{{D_{TR} }_{wired}^{i,j} \left( t \right)}}$$

Similarly, the transmission latency for wireless communication between end devices and the cybertwin-enabled edge cloud is calculated using Eq. (12),

12 $$T_{wireless}^{{\left( {i,j} \right)}} \left( t \right) = \frac{{l_{i} \left( t \right)}}{{{D_{TR} }_{wireless}^{i,j} \left( t \right)}}$$

Therefore, the total latency, which includes wired + wireless communication, can be calculated using Eq. (13), here $f_{j}$ is denoted as the CPU cycle frequency of edge cloud E_Cj_ and $C_{i} \left( t \right)$ displays the no. of. CPU cycles needed to execute the task.

13 $$T_{i,j}^{edge} \left( t \right) = T_{wired}^{{\left( {i,j} \right)}} \left( t \right) + T_{wireless}^{{\left( {i,j} \right)}} \left( t \right) + \frac{{C_{i} \left( t \right)}}{{f_{j} }}$$

#### Inter-edge cloud offloading

A hosted edge cloud’s compute work can be offloaded to an adjacent edge cloud that is proximate and can guarantee service quality if it is incapable to meet the QoS specifications of user tasks and prevent overloading. The task is generated by *E*_*Di*_ and primarily sent to *E*_*Cj*_ for execution. The adjacent E_C_ has been selected for handling the execution of tasks if the execution time ${T}_{i,j}^{edge}\left(t\right)$ exceeds the task deadline *d*_*i*_*(t)*, then the neighboring *E*_*C*_ is selected for handling the task execution.

If the neighbor node *E*_*Ck*_ is designated as the adjacent set of *E*_*Cj*_, then Eq. (14) is used to calculate the maximum data transfer rate,

14 $$D_{{TR\left( {j,k} \right)}} \left( t \right) = B_{j,k} \cdot log_{2} \left( {\frac{{P_{j,k} * g_{j,k} \left( t \right)}}{{\sigma^{2} + {\upeta }_{j,k} \left( t \right)}}} \right)$$

The communication time from *E*_*Di*_ to the initial *E*_*Cj*_, the data transfer latency between edge clouds (*E*_*Cj*_ to *E*_*Ck*_), and the task execution delay at the final edge cloud (*E*_*Ck*_) are all included in the overall latency for inter-edge cloud task offloading. This information is provided in Eq. (15),

15 $$T_{i,j,k}^{edge} \left( t \right) = \frac{{I_{i} \left( t \right)}}{{D_{{TR\left( {i,j} \right)}} \left( t \right)}} + \frac{{I_{i} \left( t \right)}}{{D_{{TR\left( {j,k} \right)}} \left( t \right)}} + \frac{{C_{i} \left( t \right)}}{{f_{k} }}$$

Correspondingly, the consumption of energy for the task offloading process is calculated using Eq. (16),

16 $$E_{i,j,k}^{edge} \left( t \right) = \frac{{P_{i,j } \cdot I_{i} \left( t \right)}}{{D_{{TR\left( {i,j} \right)}} \left( t \right)}} + \frac{{P_{j,k } \cdot I_{i} \left( t \right)}}{{D_{{TR\left( {j,k} \right)}} \left( t \right)}} + C_{i} \left( t \right) \cdot f_{k}^{2}$$

#### Task offloading to core cloud

Core cloud resources are utilized for the delay-tolerant tasks which are in need of intensive computation. Offloading tasks to the core cloud can reduce the load on edge clouds, thereby improving overall performance. Given the sufficient resources available on the *C*_*C*_, the energy consumption and execution latency have minimal impact on performance and can be disregarded. When a task is offloaded to the *C*_*C*_, the total execution latency is formulated by Eq. (17),

17 $$T_{i}^{cloud} \left( t \right) = \frac{{I_{i} \left( t \right)}}{{D_{{TR\left( {i,j} \right)}} \left( t \right)}} + \frac{{I_{i} \left( t \right)}}{{D_{{TR\left( {j,c} \right)}} \left( t \right)}}$$

where *D*_*TR(j,c)*_*(t)* defines the maximum transmission rate of data between *E*_*Cj*_ and core cloud *C*_*C*_. The energy consumption is calculated using Eq. (18),

18 $$E_{i}^{cloud} \left( t \right) = \frac{{P_{i,j } \cdot I_{i} \left( t \right)}}{{D_{{TR\left( {i,j} \right)}} \left( t \right)}} + \frac{{P_{j,c } \cdot I_{i} \left( t \right)}}{{D_{{TR\left( {j,c} \right)}} \left( t \right)}}$$

### Cache management

A portion of the task content on the end device can be cached on the edge cloud to improve the overall performance of the end-edge-cloud network. By removing the requirement for redundant task uploads and executions, task caching lowers task completion delay and energy usage. However, this storage needs to be managed well because of the restricted caching capacity. The total caching capacity of edge cloud E_Cj_ is represented as Ca_j_ for storing the content of E_D_.

Specifically, when Ca_i,j_ = 1, the task is cached on E_Cj_; else Ca_i,j_ = 0. Therefore, at each E_C_, the following constraint must be followed which is given in Eq. (19).

19 $$\mathop \sum \limits_{i = 1}^{N} {C_{a} }_{i,j} \cdot I_{i} \left( t \right) \le {C_{a} }_{j}$$

A cyber twin, acting as an intermediary agent, can efficiently manage the data and support it with task offloading. When a task offloading result is cached on the E_C_ (i.e., Ca_i,j_ = 1), the cyber twin can simplify the execution of the task by downloading the consequence directly to the E_D_ in a single step.

### Optimization problem

The proposed network architecture aims to lower energy consumption and latency to enhance user quality of service by managing the end device’s sensitive tasks. Based on the different strategies discussed, when the task is locally executed, $X_{exec}$ = 1 else it’s equal to 0, if the task offloads to E_C,_ then $X_{i,j}^{edge}$ = 1 otherwise zero. Once the task offloads to the neighboring E_Ck_, $X_{i,j,k}^{edge}$ = 1 else zero, and when the task offloads to C_C_, $X_{i}^{cloud}$ becomes 1 else 0. Every task offloading decision needs to adhere to the following condition

20 $$X_{exec} + X_{i,j}^{edge} + \mathop \sum \limits_{k = 1}^{M} X_{i,j,k}^{edge} + X_{i}^{cloud} = 1$$

Based on this condition, the energy consumption $E_{i} \left( t \right)$ and latency/task completion time $T_{i} \left( t \right)$ for the E_D_ task execution is given as

21 $$\begin{aligned} E_{i} \left( t \right) &= X_{exec} E_{exec} + X_{i,j}^{edge} \left( {1 - ca_{i,j} } \right)E_{i,j}^{edge} \left( t \right) \\ &\quad+ \mathop \sum \limits_{k = 1}^{M} X_{i,j,k}^{edge} \left( {1 - Ca_{i,k} } \right)E_{i,j,k}^{edge} + X_{i}^{cloud} E_{i}^{cloud}\end{aligned}$$

22 $$\begin{aligned} T_{i} \left( t \right)& = X_{exec} T_{exec} + X_{i,j}^{edge} \left( {1 - ca_{i,j} } \right)T_{i,j}^{edge} \left( t \right) \\ &\quad+ \mathop \sum \limits_{k = 1}^{M} X_{i,j,k}^{edge} \left( {1 - Ca_{i,k} } \right)T_{i,j,k}^{edge} + X_{i}^{cloud} T_{i}^{cloud} \end{aligned}$$

The objection solution that helps the cyber-twin to effectively allocate resources is given as

23 $${\mathbf{F}}_{{\mathbf{1}}} :X_{exec} + X_{i,j}^{edge} + \mathop \sum \limits_{k = 1}^{M} X_{i,j,k}^{edge} + X_{i}^{cloud} = 1$$

24 $${\mathbf{F}}_{{\mathbf{2}}} :\left\{ {X_{exec} ,X_{i,j}^{edge} , \mathop \sum \limits_{k = 1}^{M} X_{i,j,k}^{edge} ,X_{i}^{cloud} } \right\} \in \left\{ {0,1} \right\}$$

25 $${\mathbf{F}}_{{\mathbf{3}}} :\mathop \sum \limits_{i = 1}^{N} ca_{i,j} \times I\left( t \right) \le Ca_{j}$$

26 $${\mathbf{F}}_{{\mathbf{4}}} :T_{i} \left( t \right) \le d_{i} \left( t \right)$$

This optimization problem has been described in terms of MDP formulation where F_4_ is to ensure that the task needs to be executed before the deadline, and F_3_ ensures the overall request should not exceed the cache resources of E_C_. F_1_ and F_2_ ensure the task is offloaded using any proposed offloading strategy.

## Hybrid SOM and deep RL-based cybertwin driven joint resource allocation

**Fig. 2:**
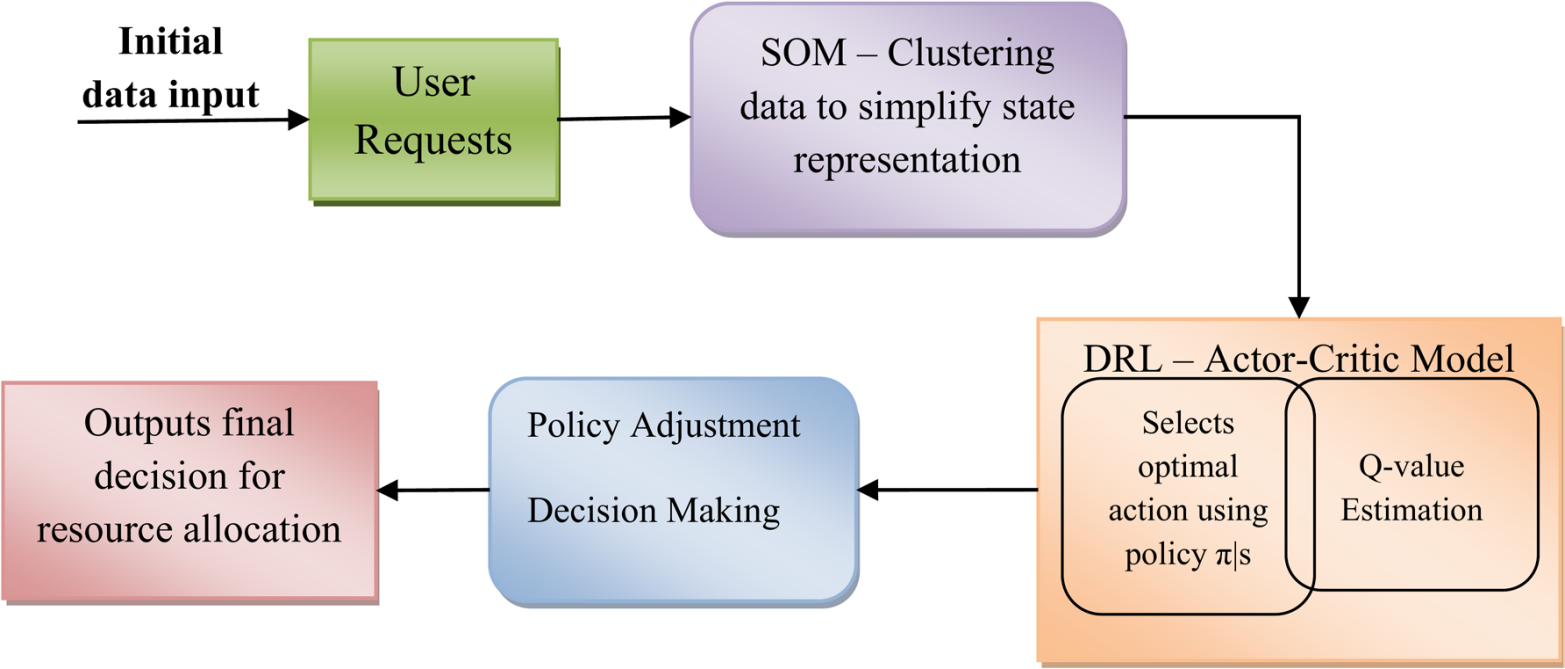
Block diagram of SOM-based deep RL.

In this proposed 6G-based cybertwin network, the hybrid model which combines Self-Organizing Map and Deep Reinforcement learning, is considered for optimized and efficient joint resource allocation for the key performance metrics such as energy consumption and latency. Figure 2 represents the block diagram of the SOM-based DRL. In order to enable real-time applications, this approach addresses the challenging task of effectively allocating resources like energy and bandwidth while decreasing latency. The hybrid technique makes use of SOM’s clustering capabilities for organizing the state space and deep RL’s decision-making abilities to identify the best path of action for allocating resources in dynamic, real-time environments.

### Self-organizing map for state clustering

SOM is inspired by the biological model of neural systems and it follows an unsupervised learning approach, where the network is trained through a competitive training algorithm. It employs dimensionality reduction and clustering techniques to move multidimensional data onto lower-dimensional regions. The input layer and the output layer are the two layers that make up SOM. It is an excellent resource for grouping and organizing complicated datasets. It can be extremely helpful in 6G-based cyber-twin networks by controlling resources, improving network management, and assisting with decision-making in real time.

Cyber-twins are digital twins of real-world objects (such as computers, people, or systems) that make resource management and control easier. SOM can be used to cluster the network states in a 6G environment, where extremely low latency and significant connections are essential. This facilitates the effective management of activities like resource allocation, latency reduction, and energy optimization. It clusters comparable network situations, enabling cyber-twins to adapt flexibly to shifting network demands and allocate resources more effectively.

A multidimensional feature vector that includes parameters like bandwidth utilization, latency, energy consumption, and user demand is used to depict the current state of the network. Equation (27) can be applied to communicate with this feature vector,

27 $$X = \left[ {E, L, B, D} \right]$$

where *E* stands for consumption of energy, *L* for latency, *B* for bandwidth use, and *D* for user demand.

Using SOM, these network states are grouped into neurons, where a distinctive collection of network states is represented by each neuron. Each neuron is linked to a certain cluster centroid by a weight vector. The SOM algorithm, adjusts the weights of the networks in order to reduce the distance between the input vector (network state) and the weight vector of the neuron, groups network states into similar clusters. Thus, using Eq. (28), the updated rule for SOM is provided,

28 $$w_{ij} \left( {t + 1} \right) = w_{ij} \left( t \right) + \eta \left( t \right)\cdot h_{ij} \left( t \right) \cdot\left( {x\left( t \right) - w_{ij} \left( t \right)} \right)$$

where *w*_*ij*_ represents the weight vector of a neuron at position (i, j); *η(t)* denotes the learning rate; the neighborhood function is denoted by *h*_*ij*_ that regulates the speed with which the winning neuron affects neighboring neurons.

SOM groups the network states according to user demand and resource availability. By simulating various network conditions, each cluster enables cyber-twins to make intelligent decisions on resource allocation. Thus, cyber-twins may efficiently regulate energy consumption, minimize latency, and improve resource allocation by clustering network states.

**Algorithm 1:**
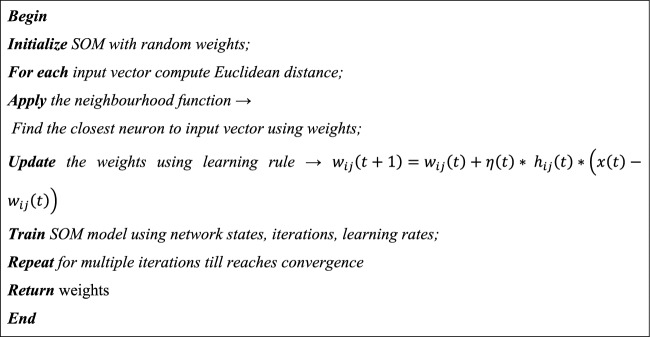
SOM clustering

### Deep reinforcement learning for resource allocation

By integrating an unsupervised learning layer into the reinforcement learning model, SOM facilitates the efficient clustering and visualization of high-dimensional state spaces. The model is better able to recognize patterns and correlations between states because of this additional layer of abstraction, which makes complex state spaces easier. SOM can be used to increase the learning process’s efficiency by decreasing the dimensionality of the input. It enhances the model’s ability to decide with higher accuracy in situations with an extensive amount of data and dynamics. Applications like 6G-based cyber-twin networks, where large-scale, real-time data processing is required, benefit significantly from this combination of features.

The most effective way to allocate resources in a cyber-twin-enabled 6G network is to use a deep reinforcement learning agent that interacts with the environment using a Markov Decision Process approach. This approach combines an Actor-Critic network model with SOM to effectively manage the complexity and variety of the network circumstances and task requirements. The Actor-Critic network model used by the deep RL framework is as follows:

Considering the present condition, the actor-network is in charge of determining the best option for action. It takes as input the reduced and clustered state representation from the SOM and outputs a policy, or the probability distribution across the action space, called π(a|s). The policy chooses the best sequence of actions in order to maximize the predicted advantages.

### MDP formulation

The problem can be described in terms of MDP with the state space, reward function, and action space as follows;

*State space (S)* The current network conditions, such as channel handling metrics, channel differences, Signal-to-Noise Ratio (SNR), and channel imperfections, are contained in the state space. It also contains details regarding the state of the task queue, the processing resources that are available, and the end device mobility patterns. In order to find patterns and lower the dimensionality of the input data, SOM is used to cluster and visualize the state space. The SOM helps with greater generalization by clustering the states, enabling the DRL agent to make decisions with greater certainty based on a simplified overview of the environment.

Equation (29) can be used to represent the state “S” at a time “t”;

29 $$S_{t} = Channel,taskqueue,Computationalresources,mobilitypatterns$$

The SOM clusters these inputs into a reduced set of representative states, *S*_*t*_, which serve as the input to the deep RL agent.

*Action space (A)* The action space consists of various possible actions; each represents a different task offloading and resource allocation scheme. The actions include options like offloading tasks to a nearby edge cloud, a cyber twin host, or a cyber twin server. Each action corresponds to a specific allocation of resources, which can be formulated as

30 $$A_{t} = \pi \left( {a{|}s^{\prime},\theta_{actor} } \right)$$

where each *A*_*t*_ denotes a particular combination of task offloading decisions, such as offloading tasks to specific E_C_, offloading tasks to C_C_ by cyber-twin, and executing tasks locally on E_D_.

*Reward function (R)* The reward function is designed to model the deep RL agent toward actions that reduce the consumption of energy and latency. Actions that result in successful task completion with lower energy use and reduced latency receive higher rewards. The reward at time ‘t’ can be given as

31 $$R_{t} = \alpha *Econ_{Total} + \beta *T_{i}^{cloud} \left( t \right)$$

Here, α and β are the weighting factors that balance the importance of energy consumption and latency. These weight factors *α* and *β* determines the priority given for the energy consumption and latency during optimization whereas α prioritizes energy consumption and *β* prioritizes latency. Therefore, the weights *α* and *β* can be dynamically fine-tuned between energy efficiency and speed based on system requirements.

The critic network evaluates the selected action by estimating the expected reward, helping the actor adjust its policy. Here the quality of the preferred action is calculated by using Q-value estimation $Q(s^{\prime}, a)$, which describes the predicted cumulative reward for taking action ‘a’ in the state (s′) and it is given in Eq. (32), where *R* indicates immediate reward, γ represented for discount factor and V(s′_t+1_) indicates the value of next state after the action.

32 $$Q\left( {s^{\prime},{ }a} \right) = R_{t} + \gamma V\left( {s^{\prime}_{t + 1} } \right)$$

The Actor network is composed of five fully connected hidden layers, which serve to process the input state (clustered using SOM) and determine the optimal action. Each hidden layer uses the Tanh activation function, which allows the network to handle both positive and negative values, helping with non-linearity and smooth transitions between decisions. After processing through the Tanh layers, the network uses Rectified Linear Unit (ReLU), a widely adopted activation function known for its simplicity and efficiency in DL models, ensuring sparse activation and mitigating the vanishing gradient problem.

**Fig. 3:**
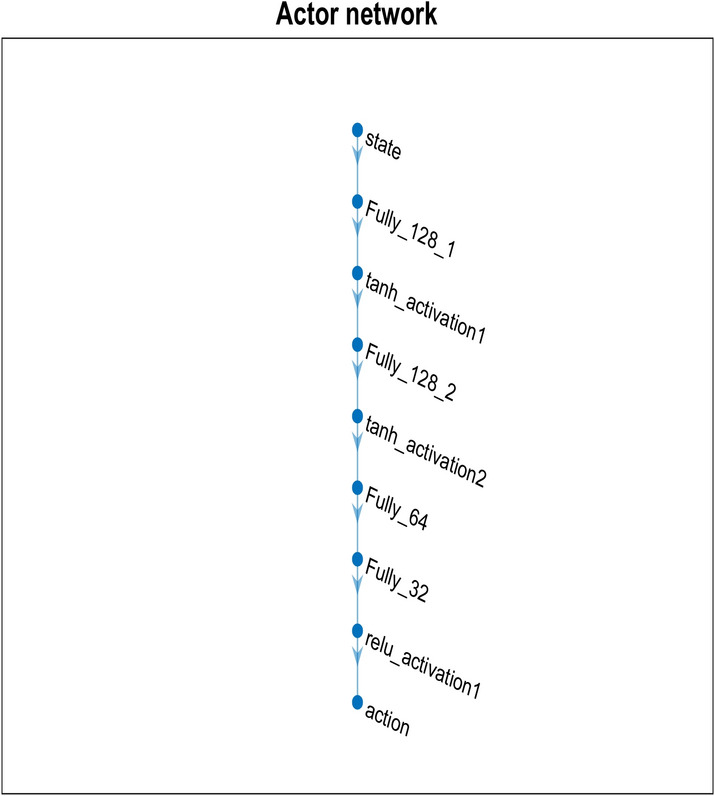
Actor-network.

**Fig. 4:**
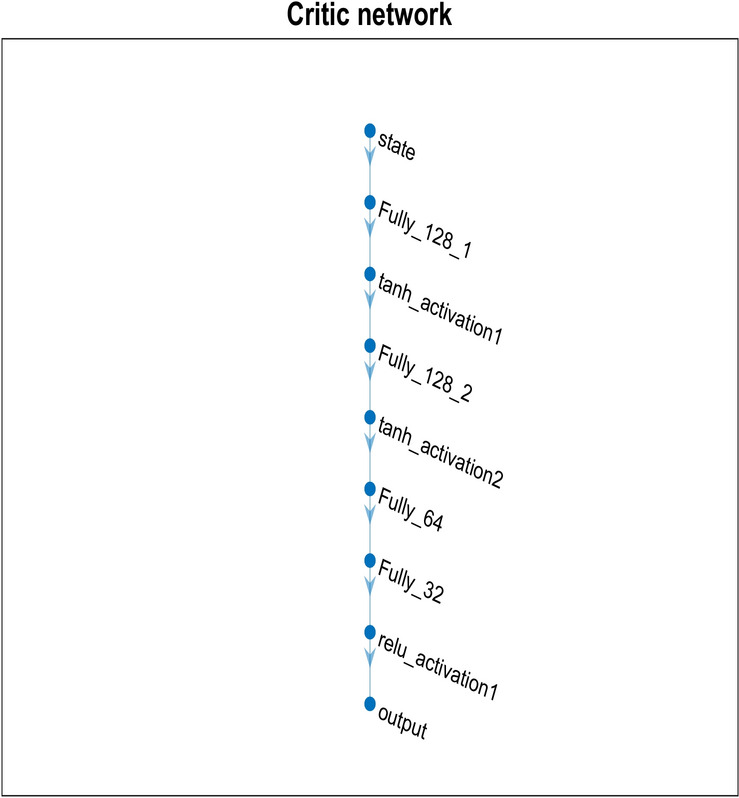
Critic network.

The Adam optimizer, a prevalent optimization technique that modifies learning rates for every parameter to speed up convergence, is used to optimize the network. With a learning rate of 0.01, the policy can be fine-tuned gradually through updates. The actor and critic network is depicted in Figs. 3 and 4.

The actor-network modifies its policy in accordance with the advantage function A(s′, a), which is provided in Eq. (33) and indicates how better the selected action was to the action in that state,

33 $$A\left( {s^{\prime},{ }a} \right) = Q\left( {s^{\prime},{ }a} \right) + V\left( {s^{\prime}} \right)$$

Equation (34) gives the policy gradient here; *η* is the actor-network learning factor, which is used to update the actor’s policy.

34 $$\theta_{actor} = \theta_{actor} + {\upeta }*\nabla_{{\theta_{actor} }} log\pi \left( {a{|}s^{\prime},\theta_{actor} } \right)*A\left( {s^{\prime},{ }a} \right)$$

The incorporation of the SOM into the MDP-based DRL model improves the agent’s understanding and adaptability in a complicated environment, resulting in more effective strategies for allocating resources.

**Fig. 5:**
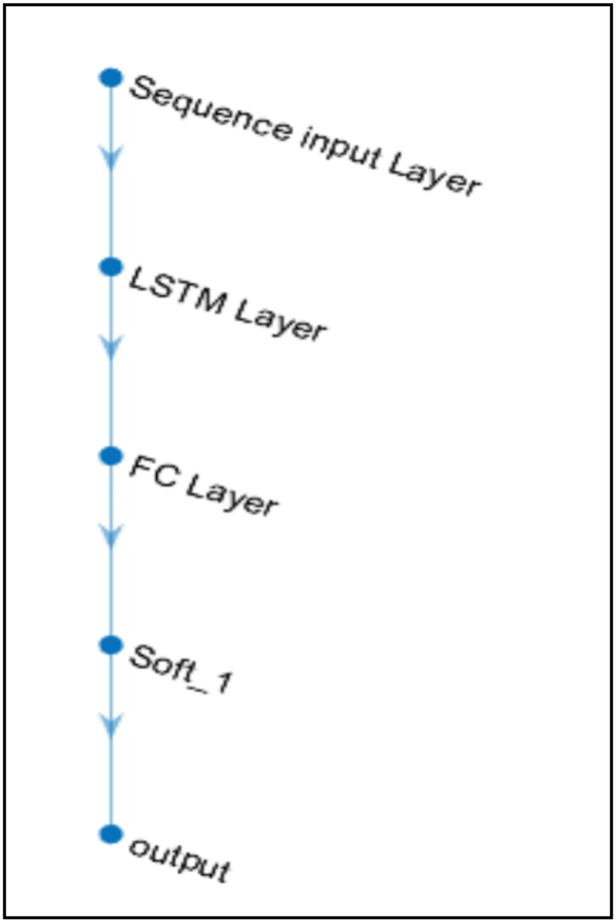
DNN layer.

The hybrid SOM-DRL model processes the state space and assists in decision-making in the dynamic, real-time 6G network resource allocation procedure through means of a deep neural network structure. This neural network, which is shown in Fig. 5, normally consists of layers like an output layer, a softmax activation function (soft_1), a Fully Connected (FC) layer, an LSTM layer, and a sequence input layer.

**Fig. 6:**
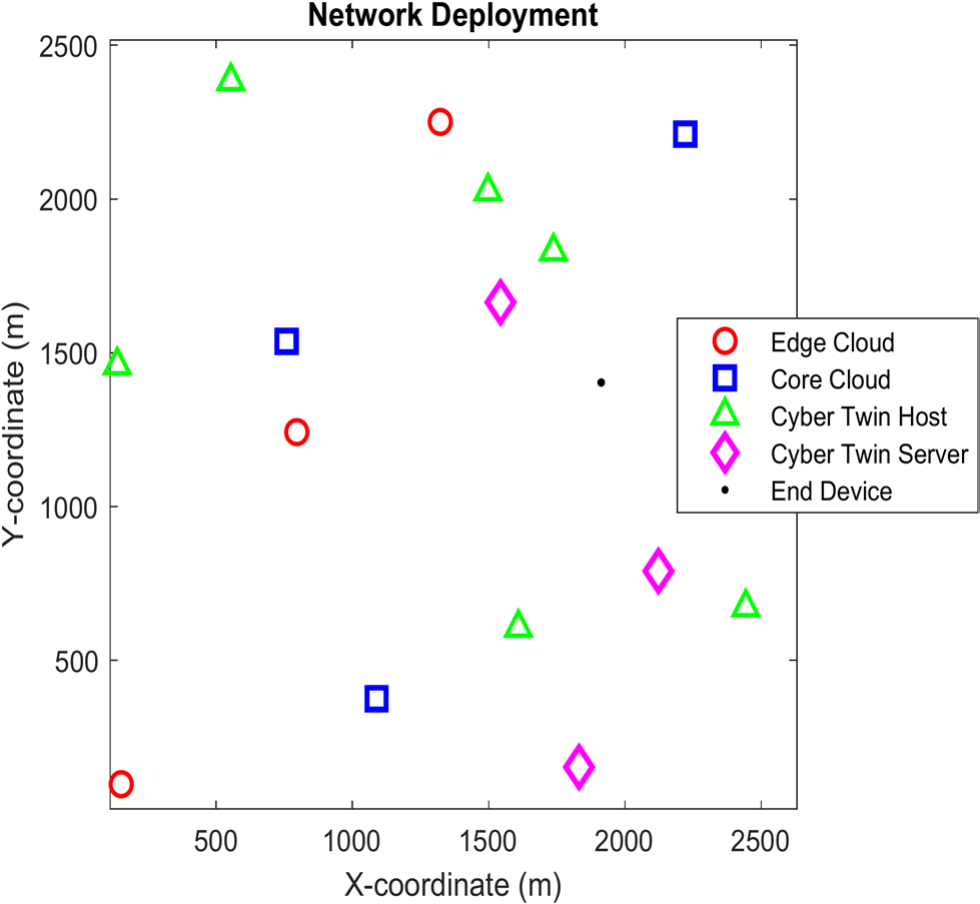
Network topology.

The sequential data that is delivered into the neural network is handled by the sequence input layer. A time series of network states, such as bandwidth utilization, energy consumption, and delay estimations, could be the input in a dynamic 6G network. States that reflect the network’s current conditions make up the data. The SOM initially clusters these states, lowering their dimensionality and organizing them in a more presentable format. These clustered states are then processed by the sequence input layer, preparing them for subsequent levels. The proposed SOM-based deep RL model’s network deployment structure is illustrated in Fig. 6.

In order for the LSTM layer to identify temporal patterns in the network data, it processes the sequential input. It aids in the process of making the best long-term decisions by keeping track of significant data from earlier time steps (such as prior network conditions). To make decisions, the FC layer takes the LSTM’s output and transforms it into a feature representation. Through training, the FC layer determines the correlations between the state input and the proper actions.

**Fig. 7:**
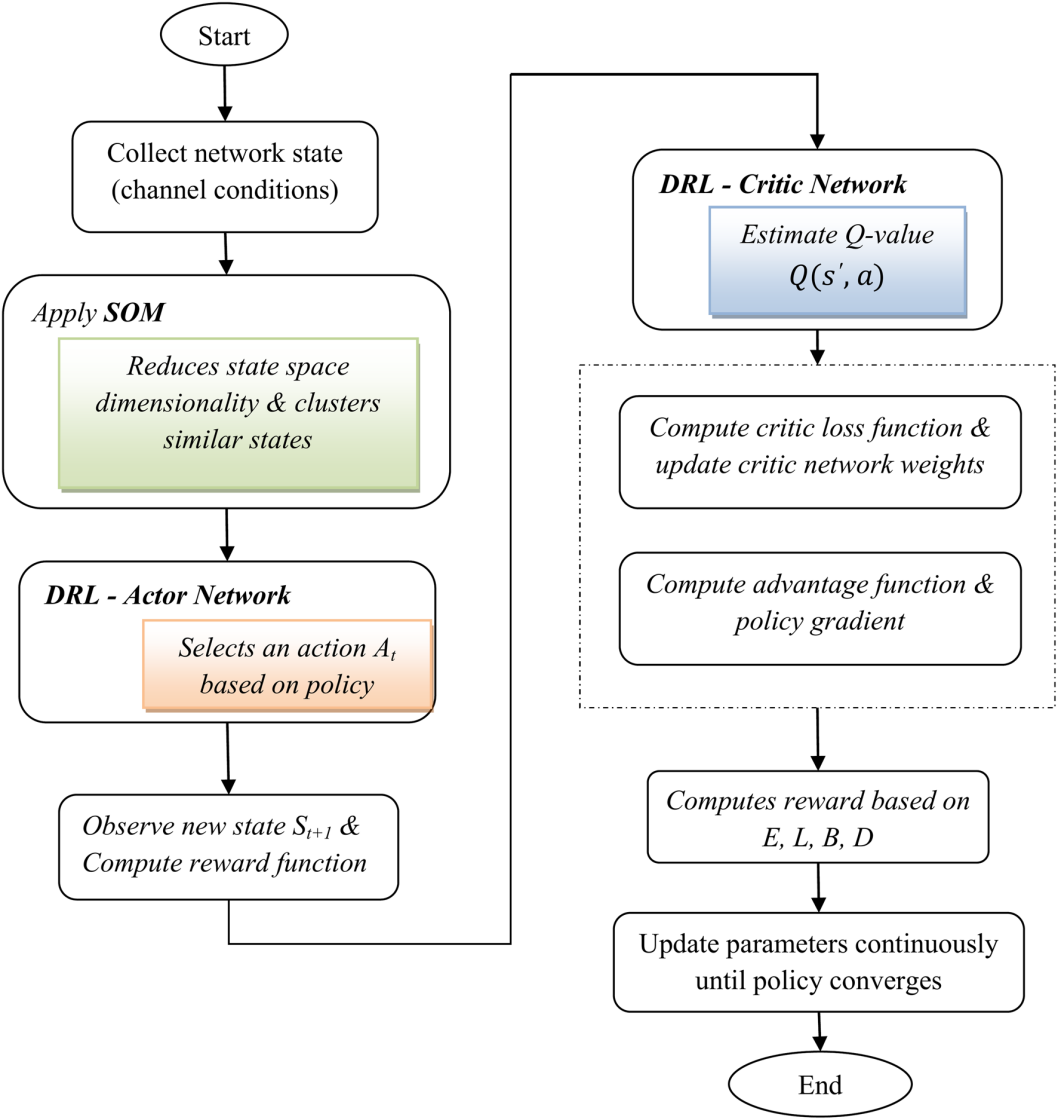
Proposed SOM-based DRL resource allocation – flowchart.

The flowchart for the proposed SOM-based DRL resource allocation is given in Fig. 7. The softmax activation function converts the output of the FC layer into probabilities for each possible action. This represents the policy of the deep RL agent that helps in a probabilistic mapping from states to actions.

**Algorithm 2:**
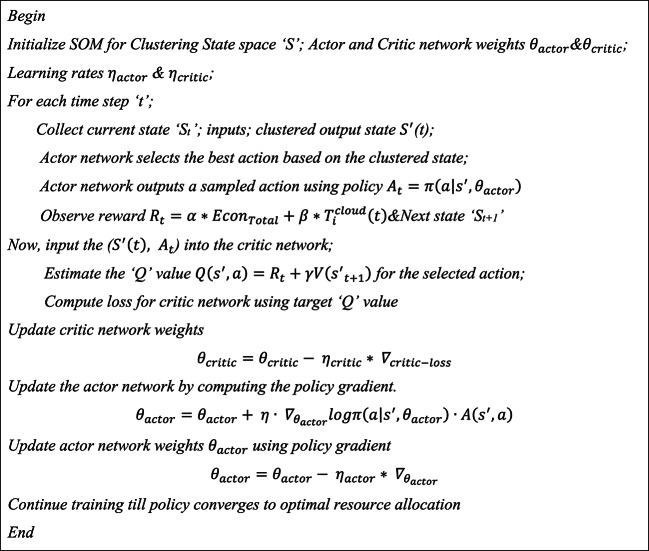
Actor-critic network model

Finally, the output layer takes the action with the highest probability (or based on exploration strategies) and executes it in the network. This action could be the allocation of bandwidth, adjustment of power levels, or offloading of tasks to the E_C_, depending on the present network state.

After taking action, the 6G network environment responds with the next state and a reward. The Critic network calculates the quality of the action by estimating the Q-value, and this feedback helps update both the Actor (policy) and Critic (value) networks. Therefore, the SOM-based actor-critic network enables the system to optimize task offloading by abstracting the state space and making better decisions, reducing both energy consumption and latency. Through continuous learning and state clustering, the system can efficiently allocate resources in dynamic and complex edge computing environments. The SOM helps the actor-critic model deal with high-dimensional input, resulting in better task-offloading performance.

## Results and discussion

A deep learning model is designed, which takes the system parameters as input and outputs the power allocation vector. SOM-based DRL architecture is utilized to allocate resources dynamically depending on the type of data and problem. The deep learning model should be trained using the prepared dataset, where input–output pairs are fed into the model, and the parameters like weights and biases are optimized to minimize the defined loss function. The trained model is estimated using a separate validation dataset to estimate its performance in generalizing to hidden data. Once the model is trained and evaluated satisfactorily, it should be integrated into the resource allocation framework. The trained model can then be used to predict the optimal power allocation vector for new sets of system parameters encountered during runtime.

Using a Random Waypoint mobility model, the end devices “E_Di_” move within the designated area at a certain speed in the direction of a randomly selected waypoint. The device may choose to rest for an arbitrary length of time after reaching the waypoint before moving on to the next destination. This model is widely used to mimic how devices move within wireless networks because it accurately depicts different movement patterns and real-world mobility conditions.

### Settings

To determine the manner in which various RL algorithms perform under various network settings, a total of 100 Monte Carlo simulations are conducted. In order to provide a varied and dynamic testing environment, 300 end devices and 4 edge clouds have been set. Through these simulations, the robustness and flexibility of the algorithms in settings with varying workloads may be evaluated. The simulation parameters and the considered actor-critic network parameters are given in Tables 1 and 2.

**Table 1:** Simulation parameters.

Parameter	Value
Number of E_D_	300
Number of E_C_	3–5
Number of C_C_	1
No. of Cybertwin Hosts	10
No. of Cybertwin Servers	4
E_C_ frequency	2.9–4.2 GHz
E_D_ frequency	16–84 MHz
CPU cycle requirements	1500–2500
Deadline of the Task	10 s

**Table 2:** Actor-critic network parameters.

Description	Actor	Critic
No. of hidden layers	5	5
Layer type	FC Activation function, tanh Layer (128X1)	FC Activation function, tanh Layer (27X1)
Type of activation function used	Optimizer Adam	ReLU
Learning rate	0.01	0.01

The agent in this SOM-based deep reinforcement learning configuration operates with a 64-bit mini-batch size and a 20,000-bit experience buffer. To direct the agent’s future reward consideration, the discount factor is set at 0.95. Training ends after 300 episodes or when the cumulative prize reaches 10,000,000. A maximum of 40 steps can be included in each episode, and a score with an average window length of 20 is used to evaluate performance. The modulation algorithm used by the system, Quadrature Phase Shift Keying (QPSK), uses 64 subcarriers.

### Result analysis for wireless communication:

In this case, the task offloading procedure involves an analysis of wireless traffic between end devices and edge clouds. The system uses SOM-based DRL to handle task offloading effectively, decreasing latency and energy consumption by making highly optimized and educated decisions. This method makes use of DRL’s adaptability and SOM’s prowess in managing complex state spaces to enhance system performance.

**Fig. 8:**
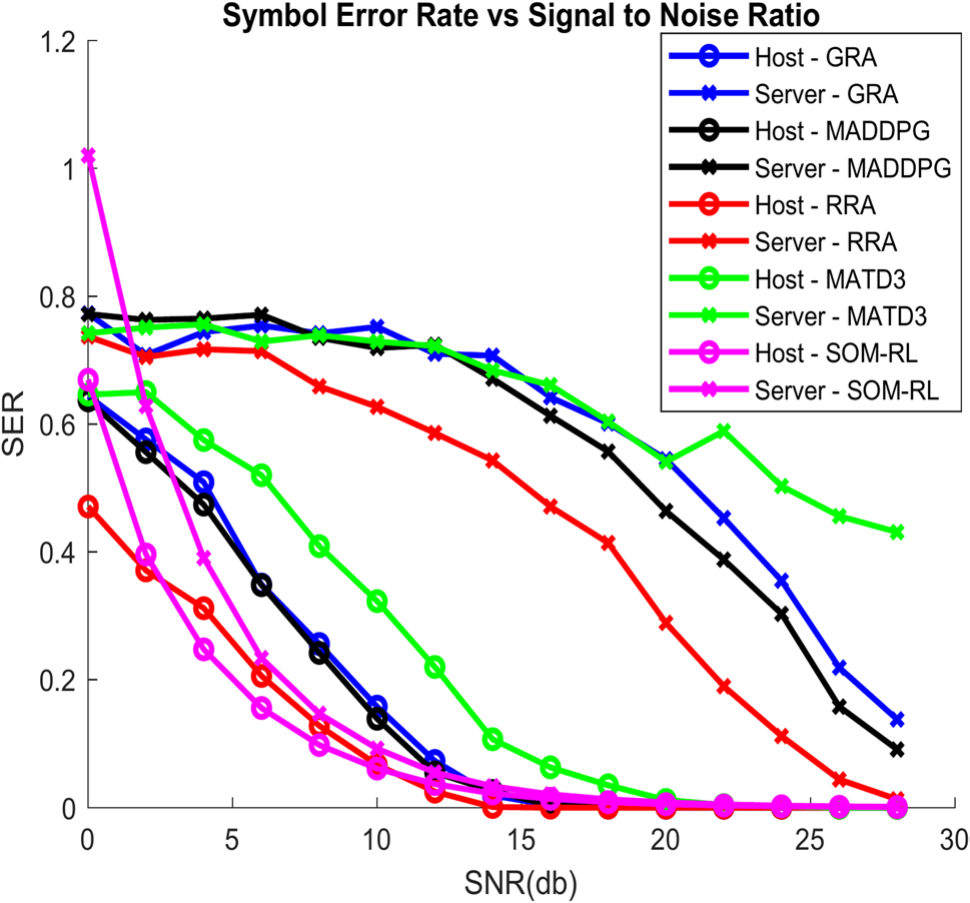
SER vs. SNR.

The Symbol Error Rate (SER) vs. SNR is displayed in Fig. 8. As SNR increases, the signal’s power increases in comparison to the noise, which lowers SER. With increased SNR, the system’s accuracy in decoding symbols improves. By minimizing interference, increasing communication efficiency, and optimizing resource allocation, the SOM-based DRL can further improve performance. Consequently, the SER may have a faster decline or yield low values when compared to conventional techniques like GRA, RRA, MADDPG, and MATD3.

The energy consumption metric calculates the amount of energy required by both edge clouds and end devices during task offloading. It considers the energy required for data transmission from endpoints to edge clouds and task processing by edge clouds.

**Fig. 9:**
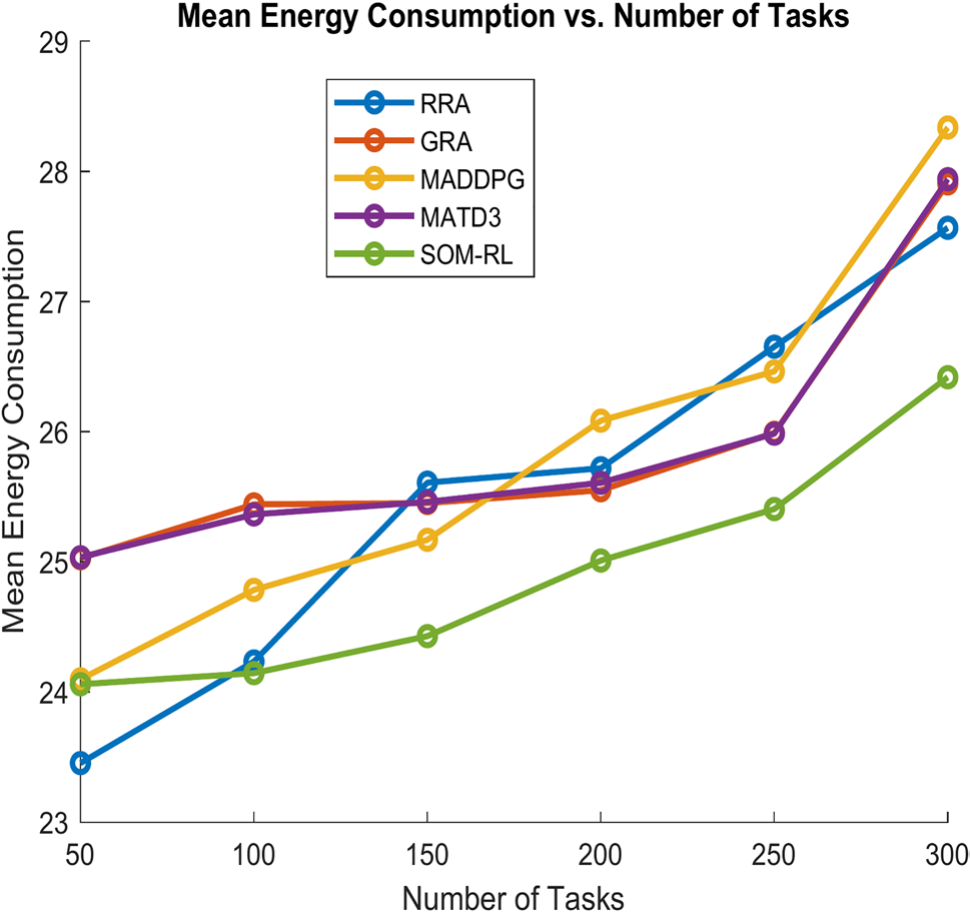
Mean energy consumption (J) vs. No. of tasks.

**Fig. 10:**
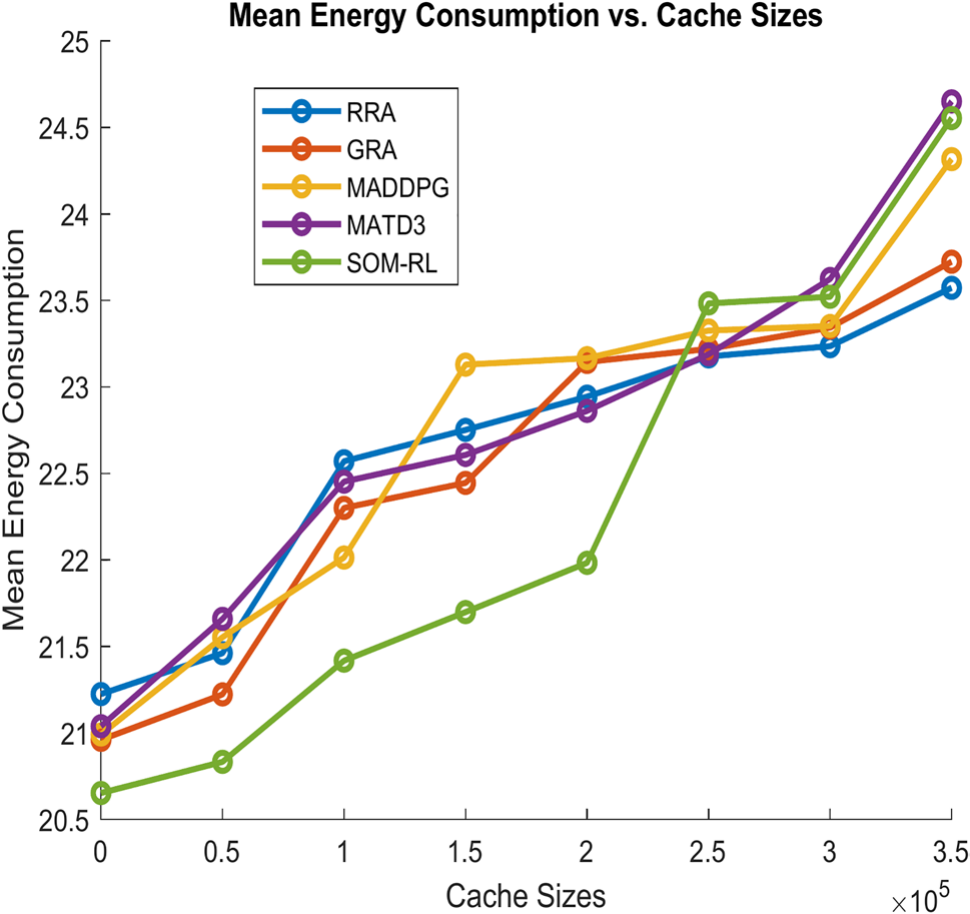
Mean energy consumption (J) vs. Cache size (b).

Figure 9 shows energy consumption with respect to the number of tasks offloading for the proposed SOM-DRL and conventional methods GRA, RRA, MADDPG, and MATD3. Figure 10 shows the mean energy consumption with respect to cache size for SOM-DRL and other conventional models. The proposed SOM-DRL consumes less amount of energy for processing with respect to the cache sizes when compared to conventional methods.

**Fig. 11:**
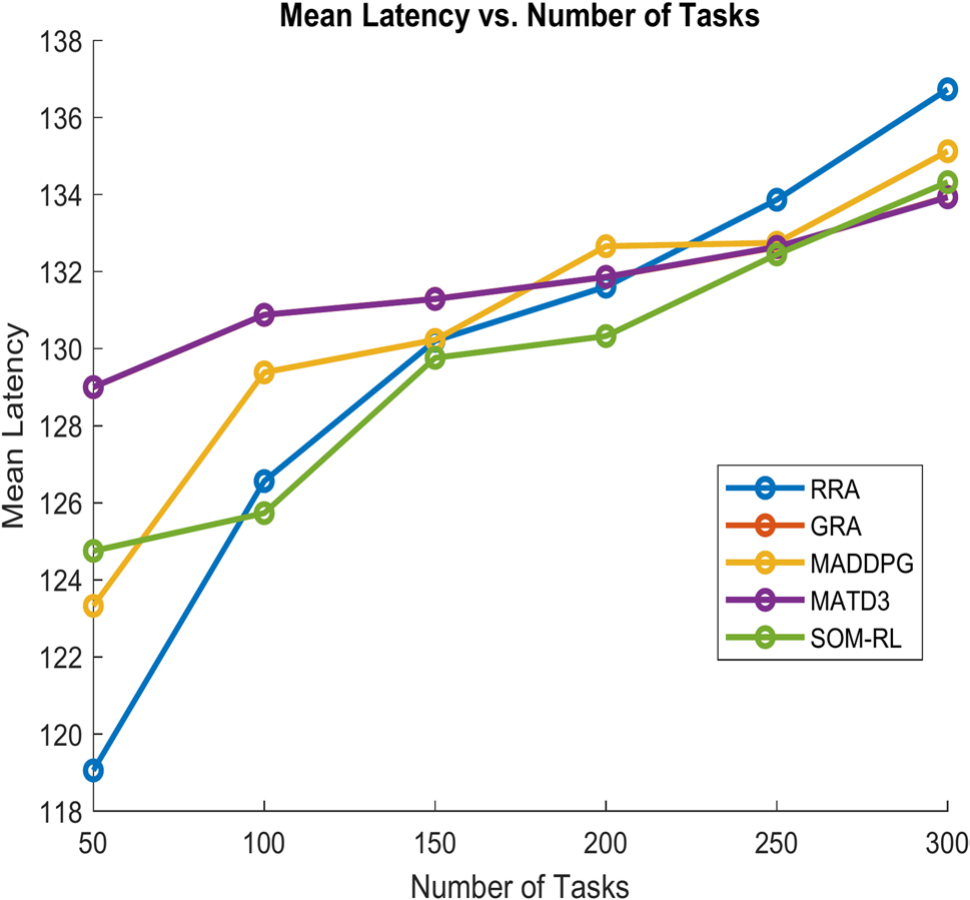
Mean latency (s) vs. No. of tasks.

**Fig. 12:**
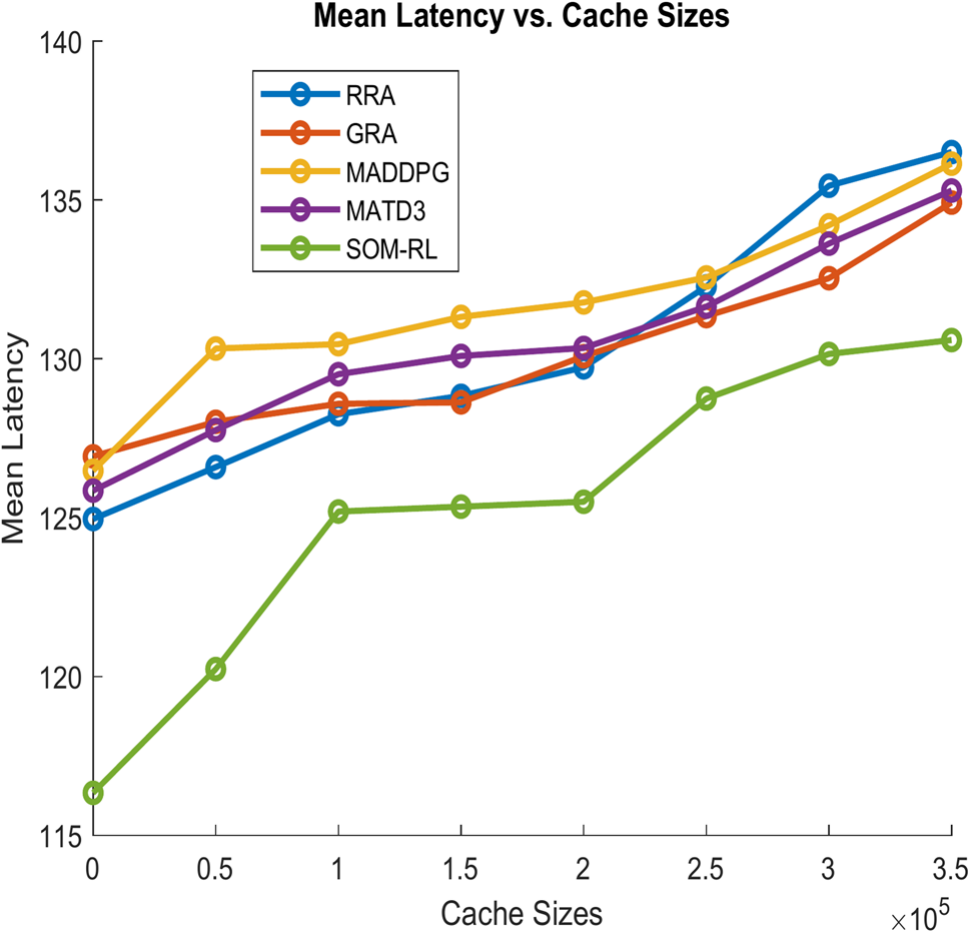
Mean latency (s) vs. Cache size (b).

Latency refers to the time delay between the initiation of a task by the end device and its completion after processing by the edge cloud. It includes the time required for data transmission and task processing. Figure 11 shows Mean Latency with respect to the number of tasks offloading for the proposed SOM-DRL and conventional methods GRA, RRA, MADDPG, and MATD3. Figure 12 describes mean latency with respect to cache size for SOM-DRL and other conventional models. Mean completion time is the average time required to complete a task or request from initial to final processing. As the cache size increases, the mean completion time usually decreases until it reaches an optimal point.

**Fig. 13:**
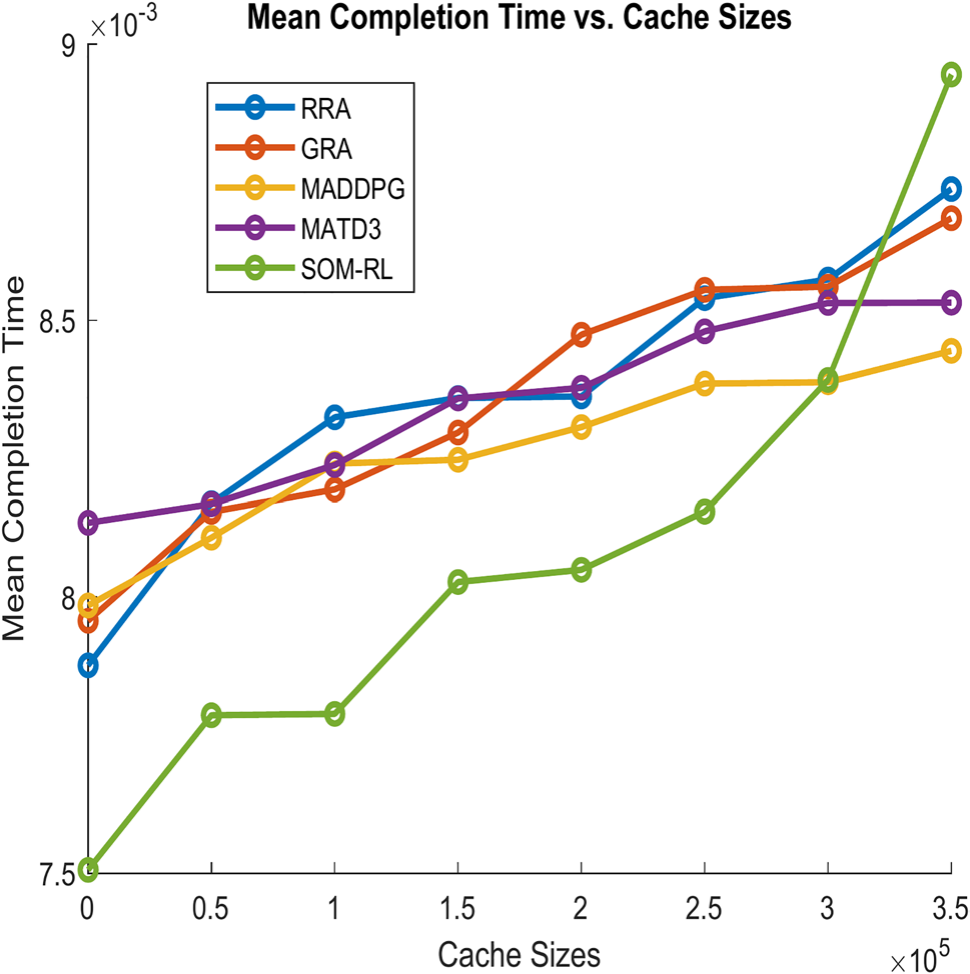
Mean completion time vs. Cache size (b).

After this point, adding more cache has less impact on improving completion time. Figure 13 shows the Mean Completion time vs. Cache Size for both proposed SOM-DRL and existing models RRA, GRA, MADDPG, and MATD3.

### Result analysis for hybrid communication (wired + wireless)

A hybrid communication system combines both wired and wireless methods to connect edge clouds and end devices. This proposed model setup uses the advantages of both communication types, such as wired communication, which provides high reliability, low latency, and consistent bandwidth, typically used for connections between edge clouds and local networks, whereas wireless communication furnishes flexibility and mobility and allows *E*_*D*_ to connect to the network without any physical constraints. The use of both wired and wireless communication allows for flexible and efficient task offloading, adapting to varying network conditions and task requirements.

The proposed SOM-based DRL using hybrid communication helps in refining the task offloading decisions by structuring the learning process and improving convergence and performance.

**Fig. 14:**
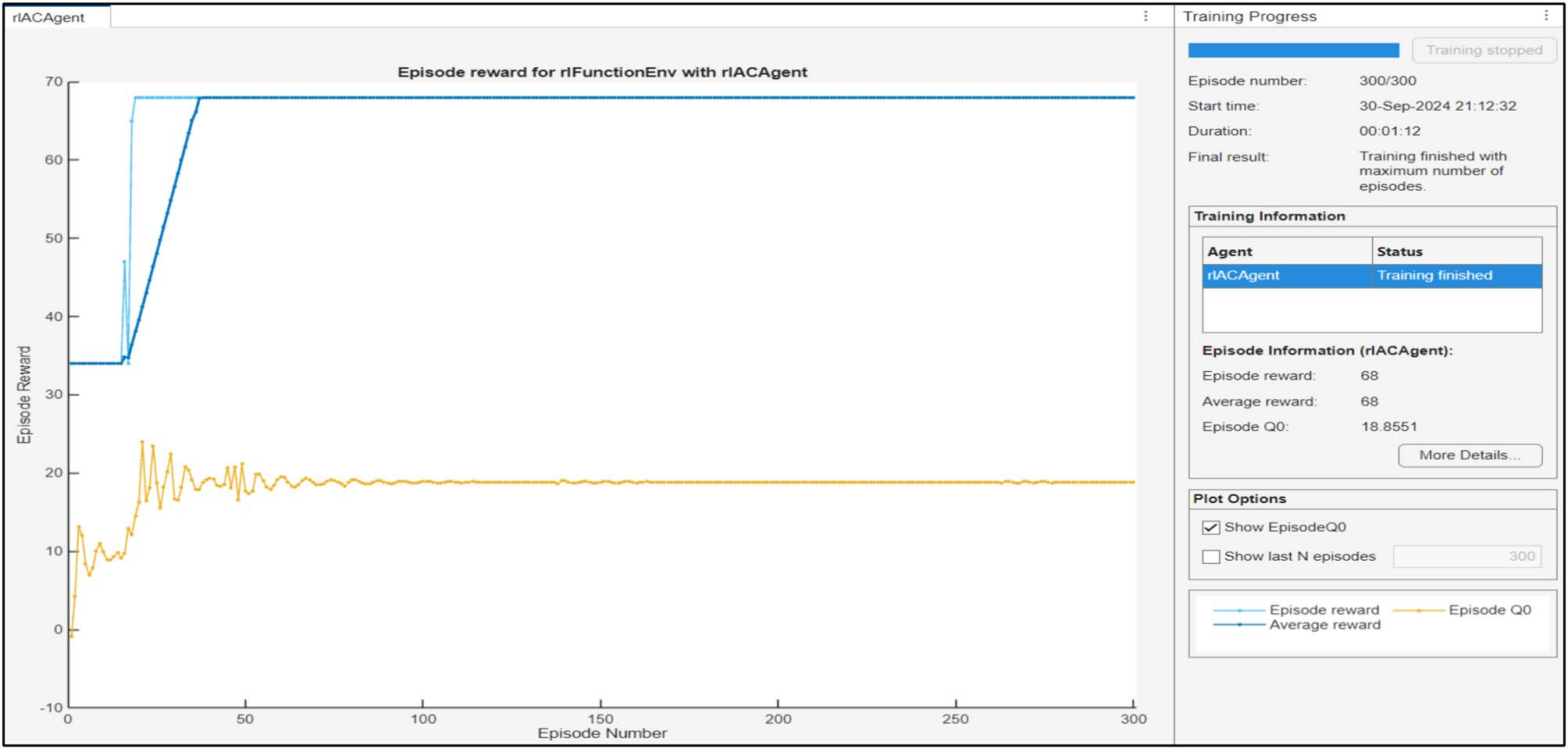
Gained reward on individual cybertwin per episode.

Figure 14 displays the reward received by a single agent deployed on a single cybertwin with three and five edge clouds. Here the episode reward obtained is 68 which is the total cumulative reward during the particular episode. The obtained value of Q0 is 18.8551 which show the confidence agent’s confidence in achieving rewards starting from the initial state of the episode that represents the initial Q-value estimate. The average episode reward that is reached in order to reach the convergence state is 68.

**Fig. 15:**
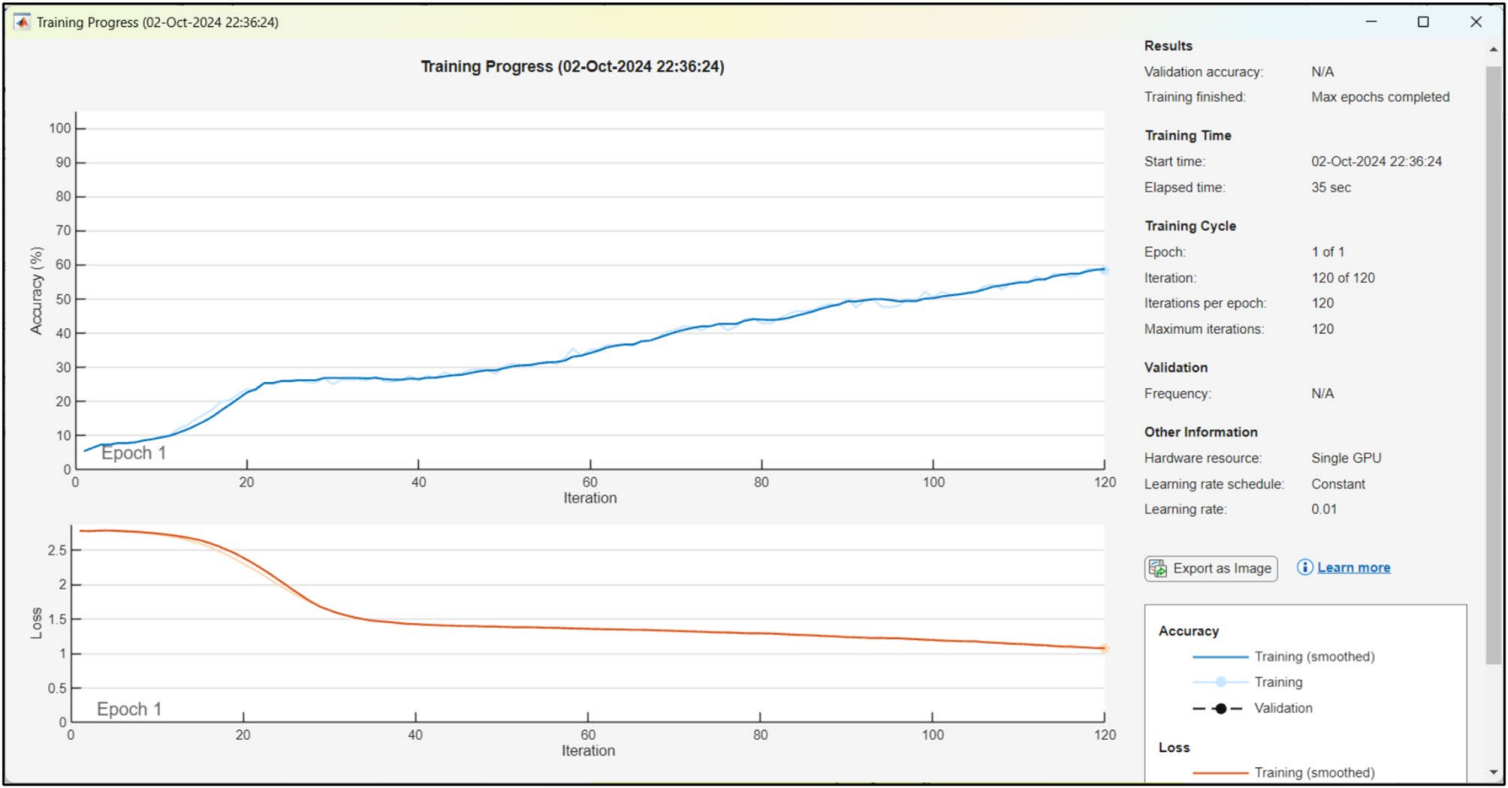
Training progress for wired + wireless links.

Based on the analysis of Fig. 15, it can be observed that the model reaches the convergence state after 30 episodes, while the attained reward is initially relatively small and varies under 0–30 episodes. Gained reward once more underwent a significant shift to a convergence condition after 80 episodes. The average episode reward that is reached in order to reach the convergence state is 68.

The training progress of a hybrid SOM-DRL model typically includes monitoring accuracy and loss, which is shown in Fig. 15. Here, the accuracy represents how strongly the model’s decisions coordinate with the optimal actions. Early in training, accuracy is low due to random exploration but increases as the model learns effective policies. Similarly, the loss measures the error in predictions by the actor-critic network. Initially high, the loss gradually decreases as the model refines its policy through feedback from the critic network.

**Fig. 16:**
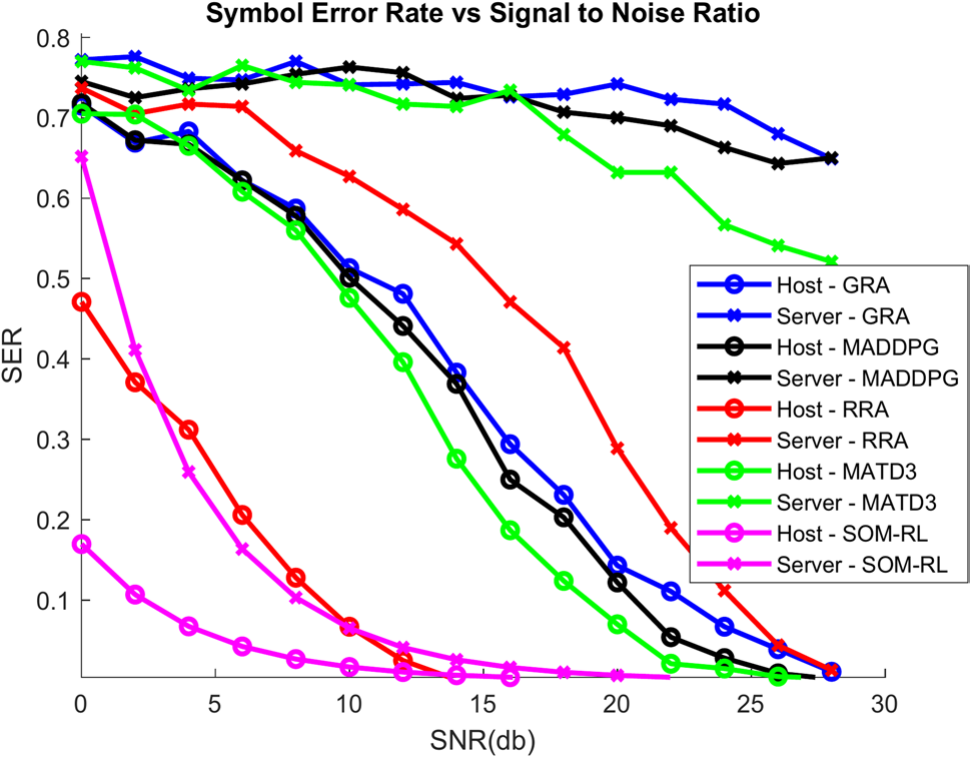
SER vs. SNR.

Figure 16 shows the SER vs. SNR; as SNR increases, the power of the signal improves relative to the noise, leading to a reduction in SER. The system’s ability to accurately decode symbols improves with higher SNR. The SOM-based DRL can enhance performance further by optimizing resource allocation, reducing interference, and improving communication efficiency. As a result, the SER might decrease more rapidly or achieve lower values compared to traditional methods such as GRA, RRA, MADDPG, and MATD3.

**Fig. 17:**
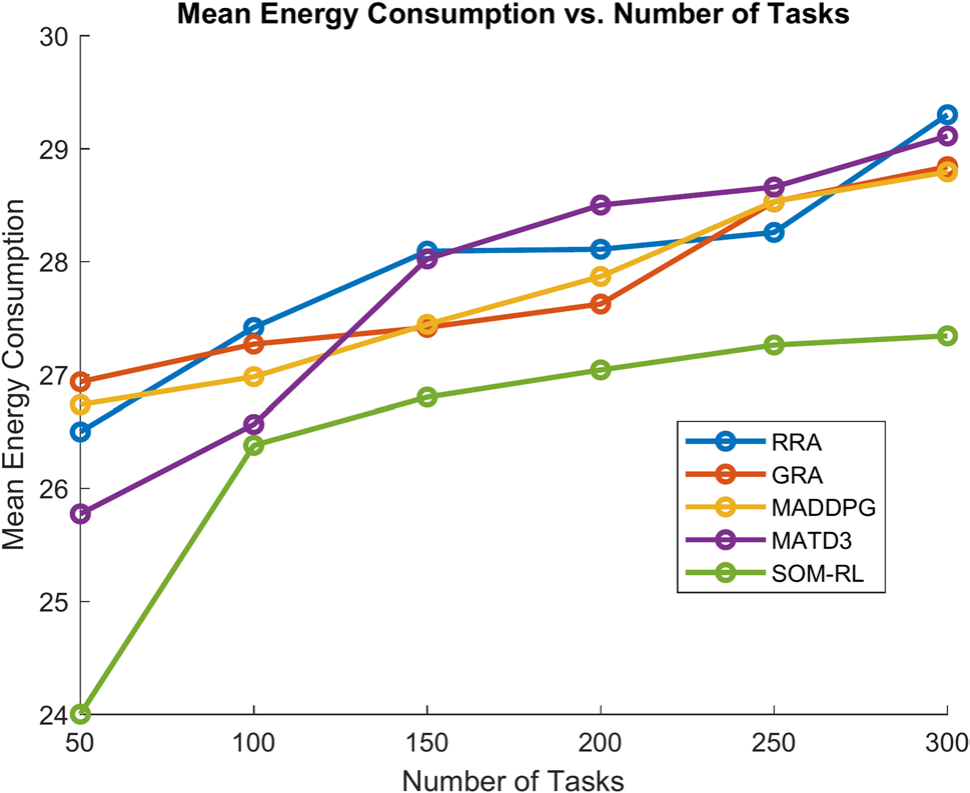
Mean energy consumption (J) vs. No. of tasks.

**Fig. 18:**
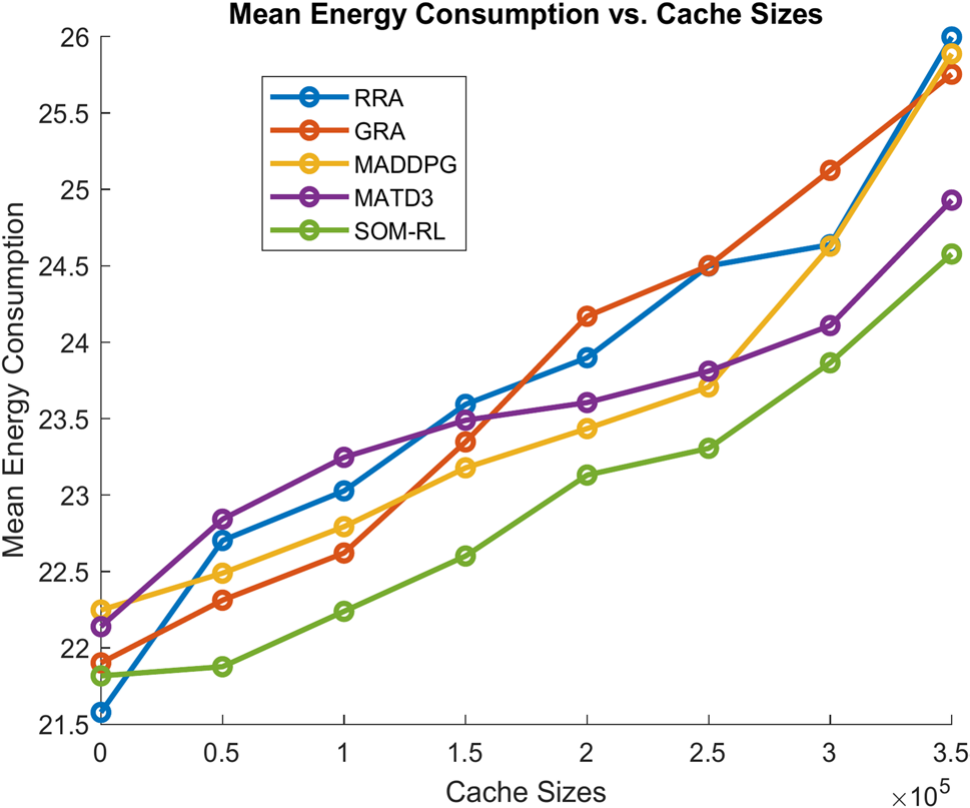
Mean energy consumption (J) vs. Cache size (b).

The energy consumption metric measured for the proposed SOM-DRL model using hybrid (wired + wireless) communication is discussed here. The energy consumption metric measures the amount of energy used by both the E_D_ and the E_C_ during the task offloading process. This includes the energy consumed in transmitting data from the end devices to the edge clouds and the energy used by edge clouds to process the tasks. Figure 17 shows energy consumption with respect to the number of tasks offloading for the proposed SOM-DRL and conventional methods GRA, RRA, MADDPG, and MATD3. Figure 18 shows mean energy consumption with respect to cache size for SOM-DRL and other conventional models.

**Fig. 19:**
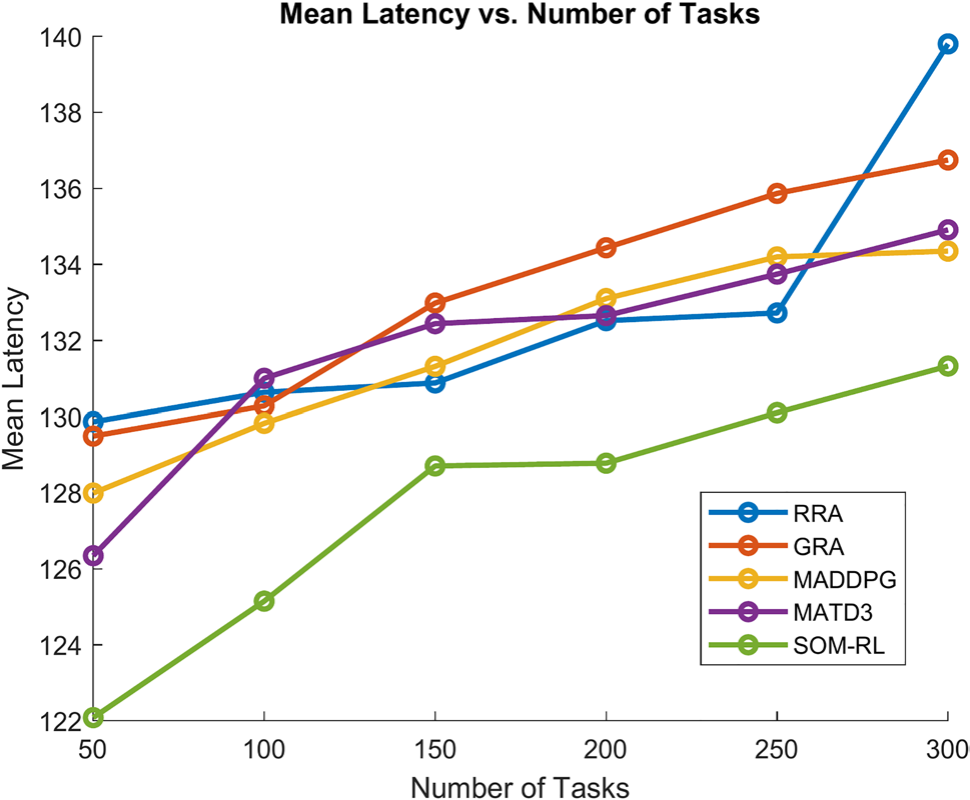
Mean latency (s) vs. No. of tasks.

**Fig. 20:**
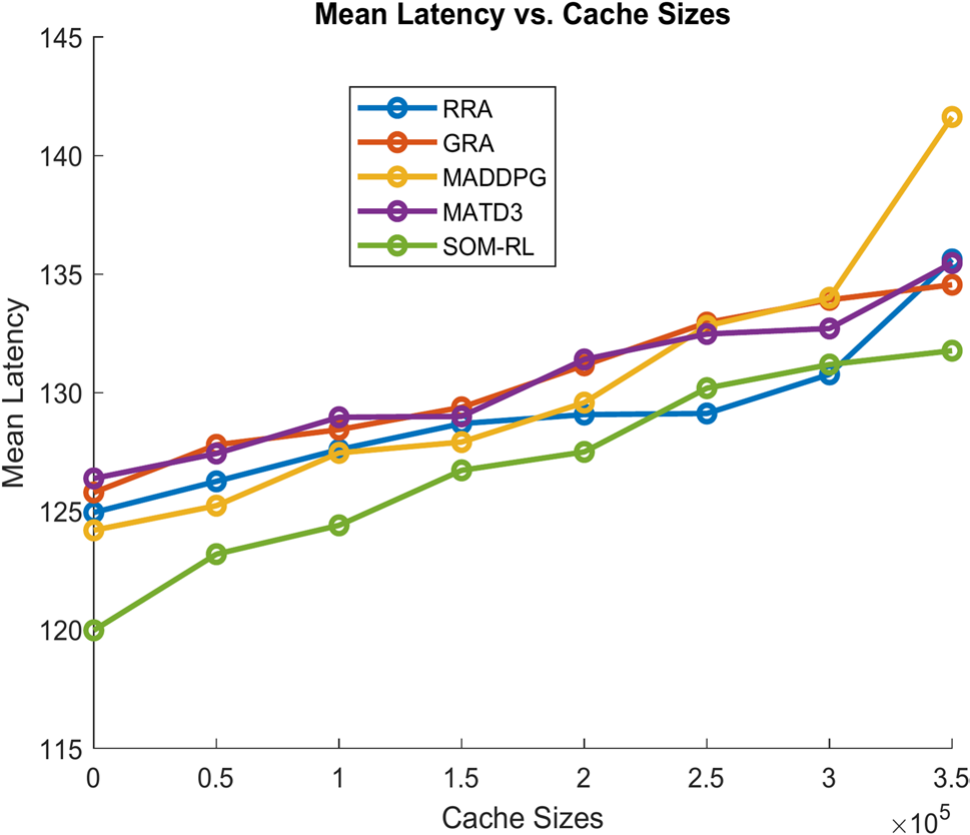
Mean latency (s) vs. Cache size (b).

**Fig. 21:**
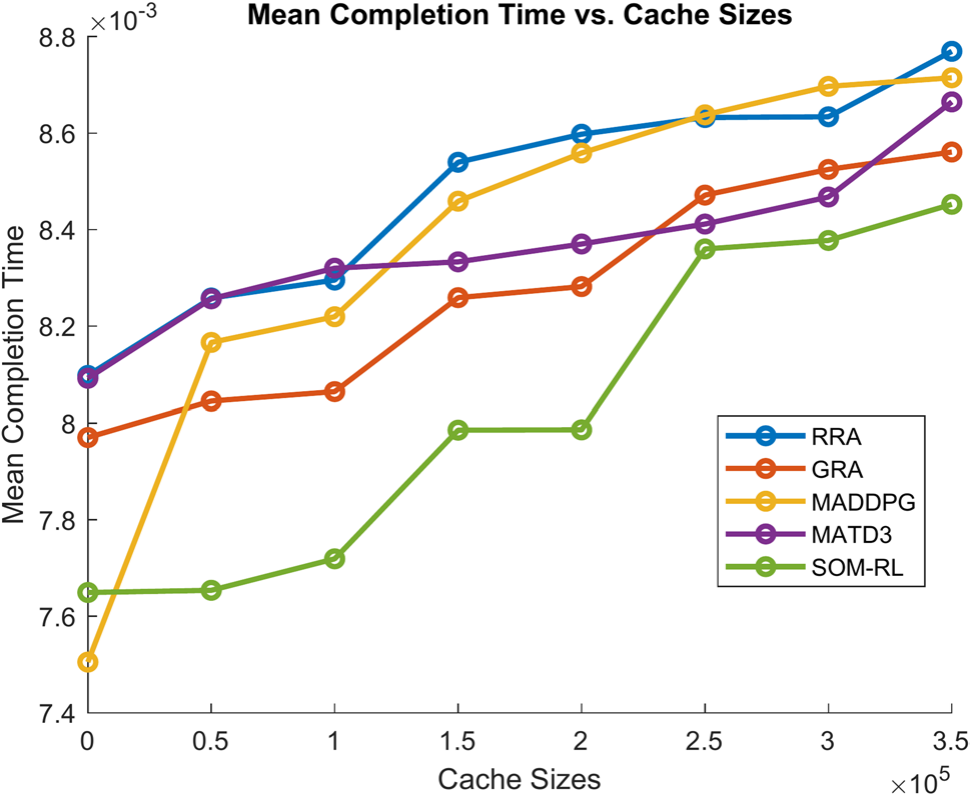
Mean completion time vs. Cache size (b).

Mean latency is one of the performance metrics measured for the analysis of the proposed SOM-DRL model using hybrid (wired + wireless) communication. Figure 19 shows the Mean Latency with respect to the number of tasks offloading for the proposed SOM-DRL and conventional methods GRA, RRA, MADDPG, and MATD3. Figure 20 describes the mean latency with respect to cache size for SOM-DRL and other conventional models. Here it can be seen that the proposed SOM-DRL obtains lower latency with respect to cache size when compared with conventional methods. The average time required to complete a task from its initial stage processing to its final stage is measured as the mean completion time. The mean completion time vs. cache size for both proposed SOM-DRL and existing models RRA, GRA, MADDPG, and MATD3 is shown in Fig. 21.

As the cache size increases, the mean completion time usually decreases until it reaches an optimal point. After this point, adding more cache has less impact on improving completion time. It can be seen that the obtained mean completion time for the proposed SOM-DRL is optimal compared to their conventional methods.

The performance of five algorithms, RRA, GRA, MADDPG, MATD3, and SOM-DRL, was evaluated based on their mean energy consumption, mean latency, and mean completion time with respect to the number of tasks. The values obtained for wireless communication for various algorithms are shown in Table 3. The SOM-DRL algorithm demonstrated the best results in terms of mean energy consumption, mean latency, and completion time, outperforming the other algorithms. SOM-DRL recorded the lowest mean energy consumption of 22.5062, mean latency of 119.954, and mean completion time of 0.00778607. In comparison, the MATD3 algorithm had a mean energy consumption of 23.6904, a mean latency of 124.684, and a mean completion time of 0.00810738.

**Table 3:** Obtained values for wireless communication of various algorithms.

Algorithm	Mean energy consumption (J) vs. No. of tasks	Mean latency (s) vs. No. of tasks	Mean ENERGY consumption (J) vs. Cache size (b)	Mean latency (s) vs. Cache Size (b)	Mean completion time
RRA	24.6331	125.065	21.5532	126.593	0.008116801
GRA	23.6035	125.0688	21.4609	128.017	0.00816916
MADDPG	23.994	126.93	21.227	130.325	0.008153399
MATD3	23.6904	124.684	21.6593	127.76	0.00810738
SOM-DRL	22.5062	119.954	20.9328	120.235	0.00778607

**Fig. 22:**
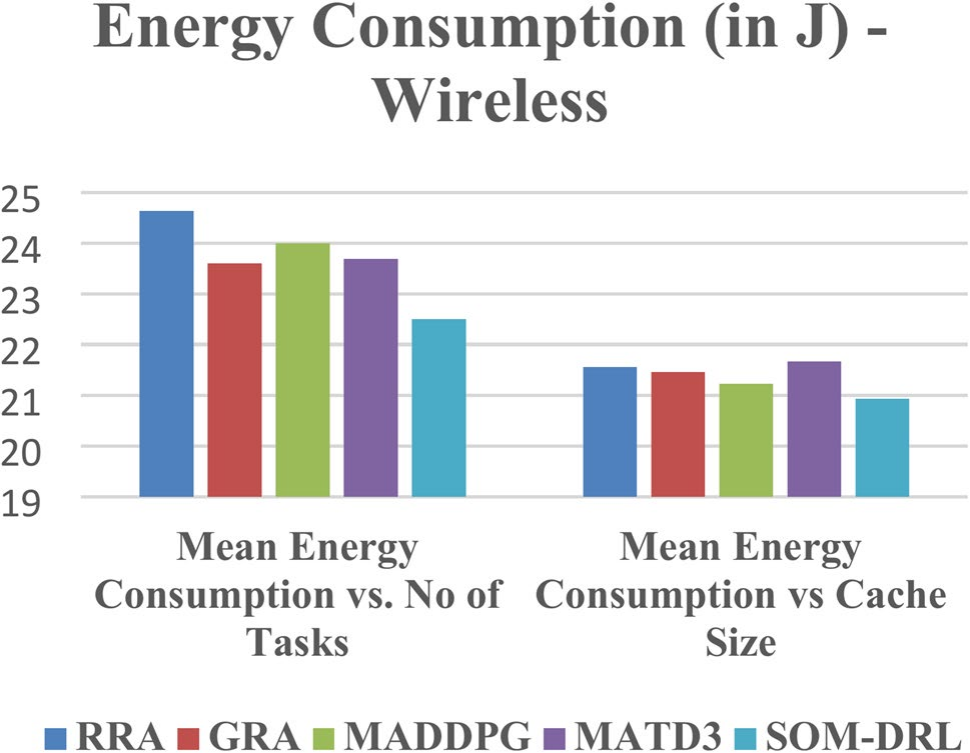
Comparison of mean energy consumption (WL).

**Fig. 23:**
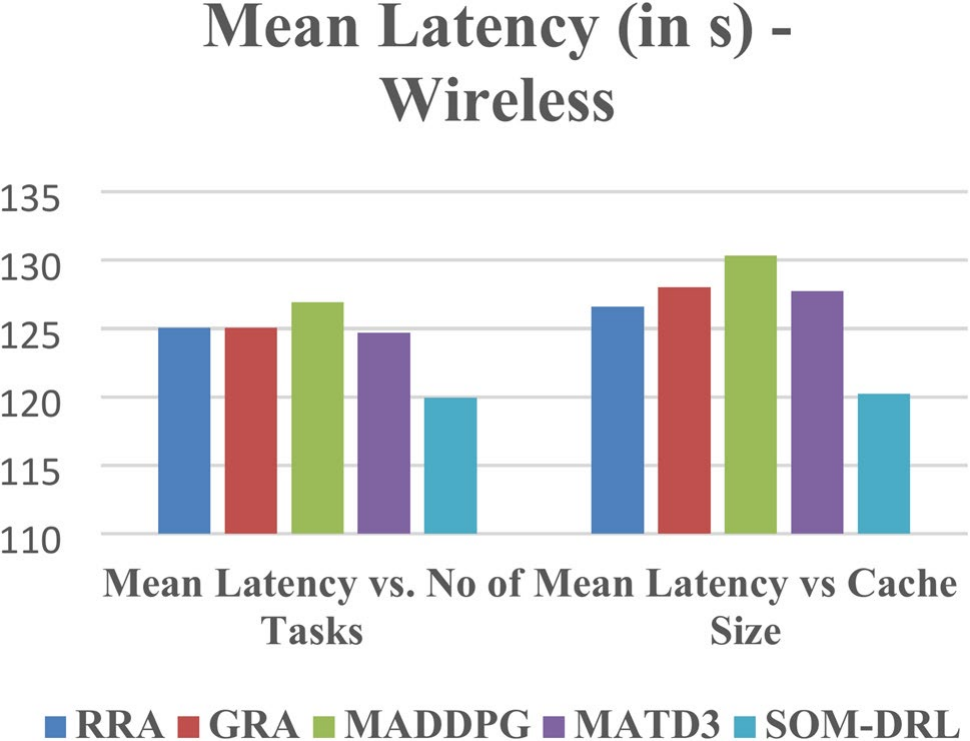
Comparison of mean latency (WL).

The percentage efficiency improvement of the SOM-DRL algorithm compared to the MATD3 algorithm in terms of energy consumption, latency, and completion time. Therefore, SOM-DRL shows an improvement of 4.98% in energy consumption, 3.79% in latency, and 3.96% in completion time when compared to MATD3. Figure 22 shows the comparison of energy, and Fig. 23 shows the latency for the proposed SOM-DRL and conventional algorithms in wireless communication. These results show that SOM-DRL is the more efficient algorithm compared to conventional methods.

The performance of the conventional and proposed algorithms, such as RRA, GRA, MADDPG, MATD3, and SOM-DRL, was assessed in terms of their mean energy consumption, mean latency, and mean completion time as a function of the number of tasks.

**Fig. 24:**
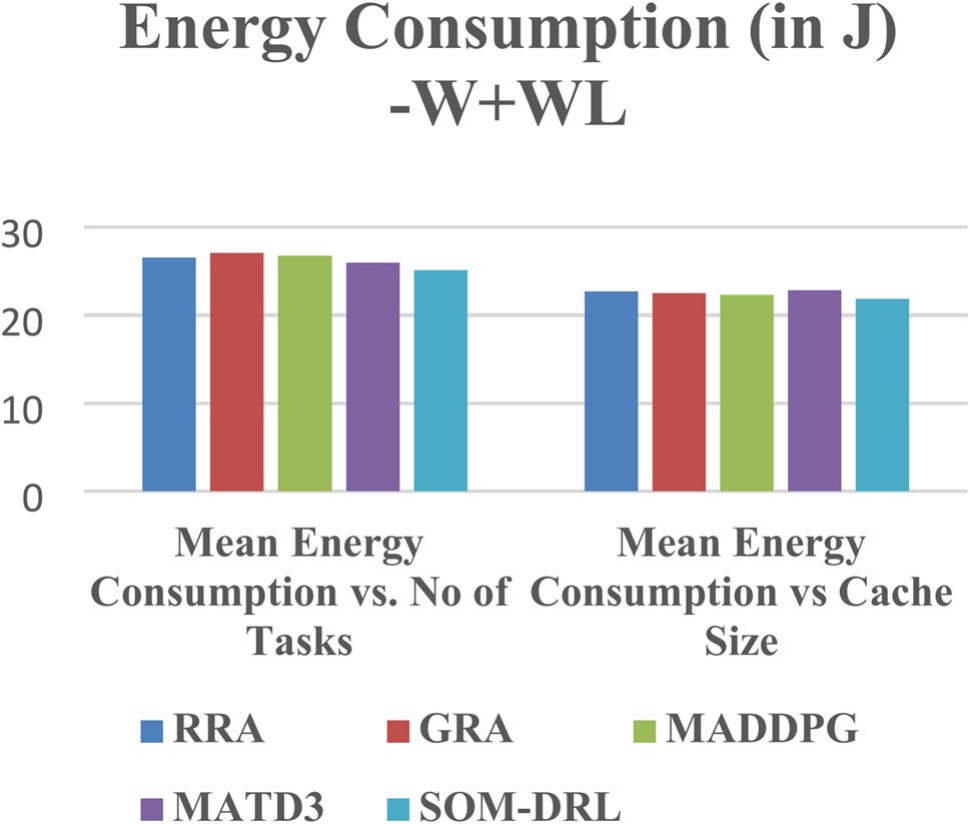
Comparison chart of energy consumption for a hybrid link.

**Fig. 25:**
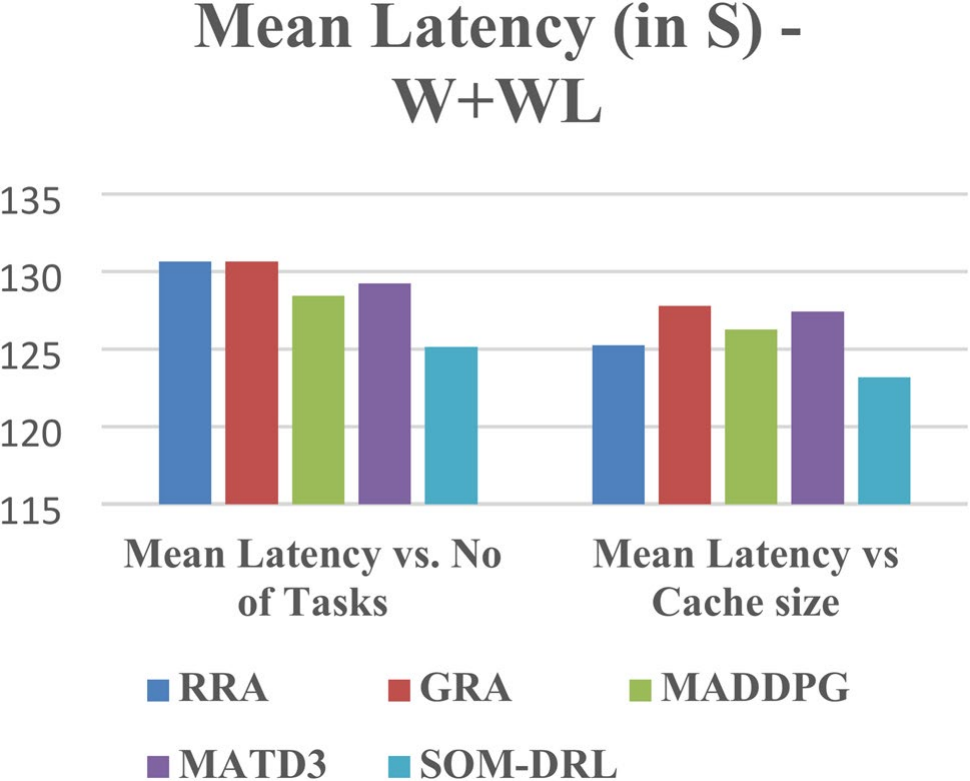
Comparison chart of latency for a hybrid link.

**Fig. 26:**
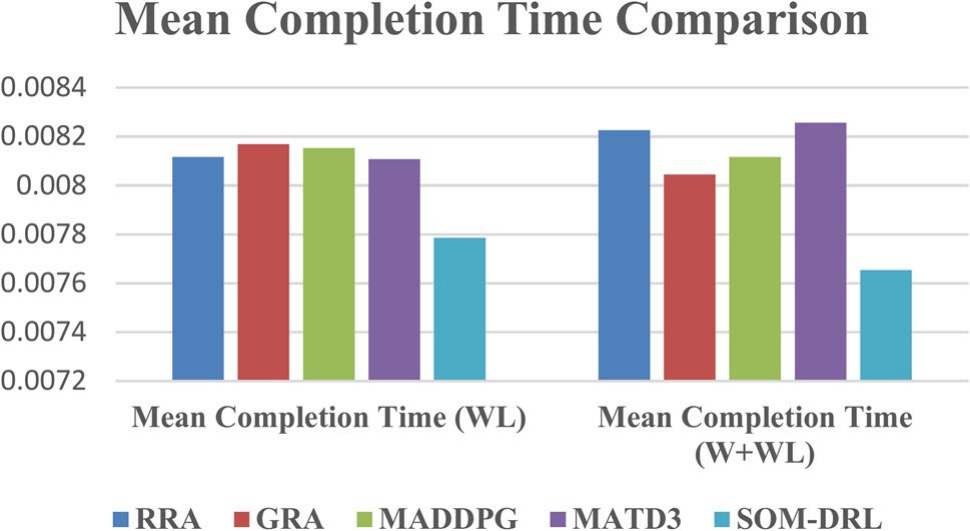
Comparison chart of mean completion time for wireless and hybrid link.

The values obtained for hybrid (wired + wireless) are shown in Table 4. Among these, the SOM-DRL algorithm achieved the best results, recording the lowest mean energy consumption of 25.0909, mean latency of 125.149, and mean completion time of 0.007654. In comparison, the MATD3 algorithm obtained values of mean energy consumption of 25.9592, mean latency of 129.25, and mean completion time of 0.008257. Figure 24 shows the comparison of energy, and Fig. 25 shows the latency for the proposed SOM-DRL and conventional algorithms for hybrid communication. Figure 26 describes the comparison of mean completion time for both wireless and hybrid communication for the proposed and other conventional algorithms. The hybrid (wired + wireless) network achieves better mean completion time for the considered existing and proposed algorithms when compared with the wireless-only network.

**Table 4:** Obtained values for hybrid communication of various algorithms.

Algorithms	Mean energy consumption (J) vs. No. of tasks	Mean latency (s) vs. No. of tasks	Mean energy consumption (J) vs. Cache size (b)	Mean latency (s) vs. Cache size (b)	Mean completion time
RRA	26.5641	130.64	22.7025	125.235	0.008226
GRA	27.0452	130.635	22.4886	127.801	0.008045
MADDPG	26.7389	128.439	22.3119	126.261	0.008117
MATD3	25.9592	129.25	22.8418	127.436	0.008257
SOM-DRL	25.0909	125.149	21.8765	123.192	0.007654

The SOM-DRL algorithm shows an improvement of 3.34% in energy consumption, 3.17% in latency, and 7.30% in completion time when compared to the MATD3 algorithm. These results indicate that SOM-DRL is more efficient than the conventional algorithms.

Therefore, the proposed model SOM clusters similar states and reduces dimensionality effectively, which enables the DRL actor-critic agent to generalize better. The actor–critic network balances the exploration and exploitation for optimized resource allocation. Hence, this proposed model adapts to changing network conditions in 6G-enabled cyber twin networks. However, for large state-action spaces, it requires high computational demand, and training time increases for larger networks.

## Conclusion

The incorporation of cyber-twin-enabled edge computing into 6G networks presents an optimistic approach to addressing the increasing demands of real-time applications with substantial data and computational requirements. There are many challenges since resource allocation is complex in dynamic edge contexts. By optimizing resource distribution in cyber-twin-enabled 6G hybrid networks, the proposed joint resource allocation technique named SOM-DRL model, effectively tackles these issues. The analysis of the results indicates that the hybrid model performs better in terms of latency and energy usage than wireless-only options. In the case of hybrid communication, the SOM-DRL algorithm outperforms the MATD3 method by 3.34% in energy consumption, 3.17% in latency, and 7.30% in completion time. This method is a significant solution for the demands of next-generation networks since it not only lowers latency and energy consumption but also guarantees secure authentication for cyber-twins. The integration of Graph Neural Networks, a more advanced artificial intelligence model, can improve the hybrid model of SOM and deep RL even further. Additionally, future research efforts can concentrate on refining this framework for extremely diverse and expansive networks.

## Data Availability

The datasets used and/or analysed during the current study are available from the corresponding author on reasonable request.

## List of symbols

E_D_ = (E_D1_, E_D2_,…E_Di_,…E_DN_): Set of End devices
E_C_ = (E_C1_, E_C2_,…E_Cj_,…E_CM_): Set of Cybertwin-enabled Edge Clouds
C_C_: Core Cloud
i: Index of end devices
j: Index of edge clouds
C_i_(t): CPU computation cycle
I_i_(t): Input data size
D_i_(t): Maximum deadline
loci(t): Device location
Dist_a,b_: Distance Parameter
f_i_: CPU frequency cycle
${\text{T}}^{{{\text{edge}}}},{\text{T}}_{{\text{i}}}^{{{\text{cloud}}}}$: Task execution latency
$${E}_{Con}$$: Energy consumption
D_TR_: Data transmission rate
P_ij_: Transmission power
g_ij_: Channel gain
η_ij_: Interference
h^m^: Rayleigh fading coefficient
σ^2^: Noise power
η_j_: Constant computing coefficient
Ca_j_: Catching capacity
$${\text{h}}_{{{\text{ij}}}}$$: Neighborhood function
${\text{V(s}}_{{{\text{t}} + 1}}^{\prime } )$: Next state value.
${\text{E}}^{{{\text{edge}}}},{\text{E}}_{{\text{i}}}^{{{\text{cloud}}}}$: Task Execution Energy Consumption
η(t): Learning rate
R(t): Reward function
γ: Discount factor
w_ij_: Weight vector
α: The weighting factor of energy consumption
β: The weighting factor of latency
